# A method to determine antifungal activity in seed exudates by nephelometry

**DOI:** 10.1186/s13007-024-01144-z

**Published:** 2024-01-29

**Authors:** Benjamin Hubert, Muriel Marchi, Joseph Ly Vu, Camille Tranchant, Łukasz P. Tarkowski, Olivier Leprince, Julia Buitink

**Affiliations:** 1 Univ Angers, Institut Agro, INRAE, IRHS, SFR QUASAV, F‐49000 Angers, France; 2 INRAE, Université de Strasbourg, UMR SVQV, Colmar, France

**Keywords:** Activity antimicrobial, Defense, Dormancy, Exudate, Seed, Tomato

## Abstract

**Background:**

One of the levers towards alternative solutions to pesticides is to improve seed defenses against pathogens, but a better understanding is needed on the type and regulation of existing pathways during germination. Dormant seeds are able to defend themselves against microorganisms during cycles of rehydration and dehydration in the soil. During imbibition, seeds leak copious amounts of exudates. Here, we developed a nephelometry method to assay antimicrobial activity (AA) in tomato seed exudates as a proxy to assess level of defenses.

**Results:**

A protocol is described to determine the level of AA against the nonhost filamentous fungus *Alternaria brassicicola* in the exudates of tomato seeds and seedlings. The fungal and exudate concentrations can be adjusted to modulate the assay sensitivity, thereby providing a large window of AA detection. We established that AA in dormant seeds depends on the genotype. It ranged from very strong AA to complete absence of AA, even after prolonged imbibition. AA depends also on the stages of germination and seedling emergence. Exudates from germinated seeds and seedlings showed very strong AA, while those from dormant seeds exhibited less activity for the same imbibition time. The exudate AA did not impact the growth of a pathogenic fungus host of tomato, *Alternaria alternata*, illustrating the adaptation of this fungus to its host.

**Conclusions:**

We demonstrate that our nephelometry method is a simple yet powerful bioassay to quantify AA in seed exudates. Different developmental stages from dormant seed to seedlings show different levels of AA in the exudate that vary between genotypes, highlighting a genetic diversity x developmental stage interaction in defense. These findings will be important to identify molecules in the exudates conferring antifungal properties and obtain a better understanding of the regulatory and biosynthetic pathways through the lifecycle of seeds, from dormant seeds until seedling emergence.

**Supplementary Information:**

The online version contains supplementary material available at 10.1186/s13007-024-01144-z.

## Background

Controlling pests that are responsible for damaging crops and plants is necessary to safeguard food security and to ensure a viable income to farmers. The vast majority of seeds undergo a pelleting treatment with chemical products requiring complex and costly industrial processes. Moreover, the environmental and public health impacts of these treatments are considerable [1, 2]. It is therefore crucial to develop more sustainable and inexpensive methods of control against plant pathogens. During evolution, plants have developed effective defense strategies. In this way, the development of alternative treatments based on nonhost resistances (NHR) that are presumed to be sustainable, are a promising lever in the fight against pathogens [3]. Thus, one promising approach would be to use and/or manipulate pre-existing defense mechanisms in plants.

The biological fitness of an organism is dependent on its ability to survive and reproduce in a given environment. Thus, reproductive organs such as flowers and developing seeds are the most valuable tissues of annual plants and the deployment of defense in these tissues represents an important evolutionary advantage [4]. The level of defense expressed by the seed will determine its persistence in the soil, together with depth of dormancy [5]. Seed dormancy is a physiological state that blocks the completion of germination of an intact viable seed under favorable conditions [6]. Primary dormancy is acquired on the mother plant and is lost gradually during after-ripening, more or less rapidly depending on the genotype and conditions of storage. Secondary dormancy corresponds to a reinduction of dormancy in imbibed nondormant seeds when they encounter unfavorable environmental conditions [7]. For example, in tomato seed heat stress during imbibition can induce secondary dormancy, referred to as thermodormancy (TD) [8].

Buried dormant seeds can remain viable in the soil for decades, where the presence of pathogens and other micro-organisms is presumed [5]. To explain the persistence of living buried seeds in the soil, the “seed defense theory” associates two types of defenses with dormancy [9]. The first type of defenses is found in the seed coat and associated with physical dormancy. It encompasses physical traits such as tegument thickness and the accumulation of certain phenolic compounds. Their role is to confer impermeability to water [10] and to act as antimicrobial compounds [11, 12]. The second defense strategy is based on the accumulation of specialized metabolites [13], peptides [14] and proteins [15] inside the seed tissues. This strategy is associated with physiological dormancy [9]. Molecular analysis gives some insight on defense responses in dormant seeds. Transcriptomes of primary and secondary dormant seeds in *Arabidopsis thaliana* showed upregulation of genes linked to defense and protection compared with their expression in non-dormant seeds [16, 17]. In primary dormant *Medicago truncatula* seeds, transcript levels of all the genes involved in the biosynthetic pathway of the phytoalexin medicarpin, an anti-fungal pterocarpan phytoalexin, were up-regulated during imbibition whereas they remained low in the same batch of seeds in which dormancy was released by dry after-ripening [18]. Likewise, the abundance of the PR10 proteins in the imbibed dormant *M. truncatula* seeds and the expression of WRKY transcription factors associated with defense against pathogens was much higher than that of non-dormant seeds. However, these studies did not investigate whether these molecules have an impact of the viability of pathogens surrounding the seeds.

Imbibition of seeds leads to the leaking or exudation of compounds that contribute in part to the formation of the spermosphere, which may play an important role in protecting seed from pathogens during dormancy and the establishment of the microbiota [19]. Indeed, seed exudates have been reported to contain defense related proteins, such as β-1,3-glucanases, vicilins, cystatins, protease inhibitors and peptidases [20, 21]. There are also numerous antimicrobial peptides in seed exudates [14, 22], some capable of inhibiting bacterial mobility [23], and others having fungicide [24] or nematicide [25] properties, or even repellent effects [26].

To better characterize the seed defenses in association with seed dormancy, a rapid, and reproducible bioassay is needed. Optical density assessed by spectrometry has been used to monitor *Fusarium oxysporum* growth in seed exudates of cowpea (*Vigna unguiculate*) [20]. However, there is no proportionality between optical density and fungal biomass [27–29]. In contrast, nephelometry is another light-based technique for measuring medium turbidity, and allows to measure the growth of micro-organisms in liquid media. This method solves the short-comings of spectrometry and conventional solid-state bioassays. The underlying principle is to measure the light scattered by suspended particles, which is then converted into Nephelometry Relative Units (RNU) that is correlated to fungal biomass [30]. Measurements of RNU over time provide growth curves that can be used to extract several quantitative parameters, such as lag phase, growth rate and overall growth (AUC, Area Under the Curve). By assessing these different growth parameters, information on the type of action of putative antimicrobial molecules is provided [30]. Nephelometry has already been used for various fungi such as *Alternaria sp*. [30, 31], *Fusarium* sp., *Penicillium* sp. [32] and the yeast *Candida* sp. [33–35].

In this study, we developed a nephelometry method to indirectly measure the defensive potential of dormant seeds during imbibition by assessing the antimicrobial activity (AA) of seed exudates of tomato *(Solanum lycopersicum* L.) against *Alternaria brassicicola* (strain Ab43). We optimized the parameters influencing the sensitivity of the assay. Using genotypes from the multi-parent advanced generation inter-cross (MAGIC) population [36], we demonstrate that there is a large genetic diversity in AA of exudates from primary dormant seeds against A. *brassicicola* growth. Similarly, exudates of seeds that are secondary dormant also show AA while exudates from germinated seeds or seedlings exhibit very strong AA. This activity does not affect the growth of a tomato host fungus, *Alternaria alternata* (strain NB100 and NB66), thus illustrating the adaptation of this fungus to its host.

## Methods

### Material and dormancy assays

Genotypes H10-205, H10-165, H10-131, H10-179 and MT-209 are derived from a multi-parent advanced generation inter-cross (MAGIC) population derived from the cross between eight parents contrasted for their fruit sizes, four with large and four with small fruits [36]. One of the parents of the MAGIC population, Cervil, was also chosen due to the deep primary dormancy of the seeds at harvest. Cervil seeds did not germinate at 20 ℃ in the dark, and 8 months of storage at room temperature (after-ripening) is necessary to release dormancy (leading to 68 ± 2% germination). The plants were grown in a greenhouse in Angers between November and July 2021. The daily mean/maximal temperatures were on average 21.2 ℃/28.8 ℃. Mature red fruits were harvested from the 2nd to the 8th truss. Seeds were extracted from fruits by incubating the locular tissues for 1 h under gentle shake at room temperature in an Erlenmeyer with 100 mL of solution containing 40 mg of pectolytic enzymes (Lafazym CL^®^, Laffort, France). Seeds were extensively washed with water to remove remnants of fruit tissues. They were blotted dry on a filter paper then rapidly dried under an airflow at 43% RH at room temperature and subsequently stored at − 80 ℃ to maintain primary dormancy, which was necessary to perform all the experiments. No significant difference in germination percentage after 5d of imbibition was observed throughout the experimental time frame that lasted for 6 months. Seeds were warmed to room temperature before use. Secondary dormancy was induced by incubating 60 seeds in 2 mL of water at 35 ℃ in the dark. Dormancy release was performed by a 6-d stratification treatment at 4 ℃ in the dark in the presence of 30 mM KNO_3_ or by 8 months of storage at room temperature [37]. To test for germination, 60 seeds were imbibed with milli-Q water at 20 ℃ in the dark and germination was scored by the presence of an emerged radicle of 2 mm. Germination percentage was recorded daily after each exudate preparation.

The Ab43 strain of *A. brassicicola* was isolated from *Raphanus sativus* seeds [38]. The strain NB66 and NB100 of *A. alternata* was respectively isolated from tomato stem and potatoes leaf [39].

### Production of seed exudates

To produce exudates, 15–60 seeds, 60 germinated seeds or seedlings were imbibed with 2 mL sterile milli-Q water (Milli-Q^®^ Reference A + Water Purification System, Millipore SAS, Molsheim, France) in 30 mm diameter sterile glass Petri dish in the dark at 20 ℃ for the indicated period of time. To compensate for the water absorption by the growing seedlings, sterile water was added to maintain the liquid volume constant at 2 mL. After the indicated period of incubation, the exudates were collected and sterilized using a CHROMAFIL Xtra PA-20/13, 0.20 µm filter (Macherey Nagel GmbH & Co. KG, Dueren, Germany), aliquoted, frozen in liquid nitrogen and stored at − 80 ℃ before use. They were retrieved by slow thawing on ice in the dark.

### Nephelometry

*A. brassicicola* and *A. alternata* were grown at 25 ℃ in the dark respectively on potato dextrose agar medium (Becton Dickinson, Franklin Lakes, NJ, USA) and on potato carrot agar medium (HiMedia Laboratories, Einhausen, Germany). For inoculum preparation, conidia were collected from 7–8 d-old solid cultures by adding sterile milli-Q water and then gently scraping the agar plates. They were then counted in a Thoma’s chamber (Glaswarenfabrik Karl Hecht, Sondheim vor der Rhön, Allemagne, 0,05 × 0,05 mm, depth of chamber 0.100 mm) and the conidial suspensions were diluted in sterile milli-Q water at the indicated concentrations. To a 96-well plate, 300 µl of one of the following solutions were added in triplicates: solution a) potato dextrose broth and inoculum solution (90:10 v:v); solution b) potato dextrose broth, inoculum solution, exudate (80:10:10 v:v) and solution c) potato dextrose broth and exudate (90:10 v/v). Fungal growth was recorded using a nephelometric reader at 635 nm (NEPHELOstar Galaxy, BMG Labtech, Offenburg, Germany) according to Joubert et al. [30] with the following modifications: run time 66 h with 20 min stepwise measurements at 20 ℃.

### Analysis of growth data

Data were analyzed using Omega MARS software version 3.42 R5 (BMG Labtech, Offenburg, Germany). A correction of the background noise generated by the exudates was carried out using the signal from solution c. If the readings of solution c were too high, indicating a potential bacterial contamination, the experiment was cancelled. Slope, maximum slope corresponding to the inflection of the growth curve, time to reach maximum slope and area under the curve were calculated by the software. The lag phase was calculated as the X-intercept of the regression line obtained during the exponential growth phase. To estimate the impact of the exudate on fungal growth and allow comparisons between plates, a normalized growth was calculated as the ratio of the AUC with exudate over the AUC without exudate.

### Statistics

Data are based on a minimum of three technical repetitions and were repeated several times on different seed samples from the same batch and different conidia preparations. The number of biological replicates is indicated in each legend and ranged between two and nine, except for the kinetic data for which each individual datapoint represents three technical replicates from one biological replicate. Statistical tests were carried out using SigmaPlot 13.0 software (Systat Software Inc., Delaware, USA). Normality test (Shapiro–Wilk) and equal variance test were first performed. To test the significance of the effect of the exudate on fungal growth a two tailed t-test was performed using raw nephelometry data. When the normality test failed, a Mann–Whitney test (*p* = 0.05) was performed. To test the significance between treatments or genotypes, an ANOVA test (*p* < 0.05) was performed on the normalized growth followed by a pairwise multiple comparison using the Holm-Sidak method (*p* < 0.05). When the normality test failed, a Kruskal–Wallis test (*p* < 0.05) followed by the Dunn method (*p* < 0.05) was used. The corresponding tests are indicated in each figure legend.

## Results

### A quantitative measure of AA in exudates: inhibition of fungal growth using nephelometry

**Fig. 1:**
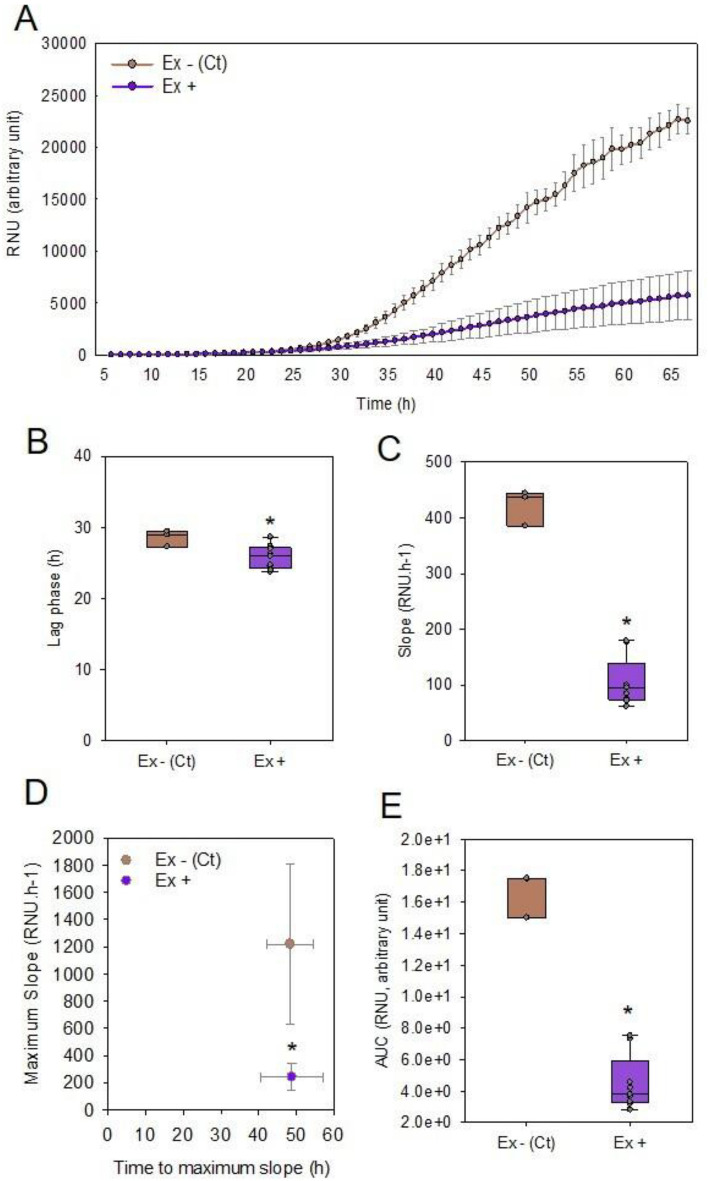
Effect of primary dormancy seed exudate on the growth of *A. brassicicola*. **A** Growth curve of *A. brassicicola* at 10^3^ CFU/mL with (Ex +) and without (Ex -; control) exudates from seed genotypes Cervil. Data represent the average of RNU ± SD. **B** Lag phase calculated as the X-intercept of the regression line obtained during the exponential growth phase. **C** Average slope. **D** Maximum slope projected as a function of time to reach maximum slope. **E** Area under the curve. Points in the box plots corresponds to three technical replicates per biological replicate (n). Control (Ex -), n = 1; Cervil (Ex +), n = 3. A star indicates a significant difference from the control (Mann–Whitney test, p < 0.05). AUC, area under the curve, RNU, relative nephelometry units

To investigate if seed exudate influences growth of *A. brassicicola* (strain Ab43), the genotype Cervil was chosen as these seeds exhibit deep primary dormancy at harvest. The addition of seed exudate collected over a 5 d imbibition period in water to a liquid medium containing conidia of *A. brassicicola* strongly decreased the fungal growth (Fig. 1A). From the growth curves, the following parameters were derived to describe and quantify the fungal growth inhibition: the lag phase, the slope (representing the growth rate), the inflexion point representing maximum growth rate and the area under the curve (AUC) that integrates all these parameters. The presence of the seed exudate induced a slight decrease of the lag phase from Cervil exudate from 28 to 26 h (Fig. 1B). A large reduction in the slope of the curve with Cervil exudate from 422 to 105 RNU/h was observed with the seed exudate compared to the control without exudate, representing 75% of reduction of the fungal growth induced by the exudate (Fig. 1C). The maximum fungal growth rates without or with exudate in the medium were 1220 and 241 RNU/h, respectively, and these values were reached at 49 h of incubation for both conditions, indicating that the exudate strongly influenced the growth potential of Ab43 (Fig. 1D). Likewise, the AUC was 1.67^9^ and 0.449^9^ RNU without and with exudate (Fig. 1E) representing 27% of the control without exudate. Since AUC integrates the growth pattern of the fungus, we determined the ratio AUC with exudate over the AUC without the exudate as a normalized growth to quantify the antimicrobial activity of the exudate.

### Modulation of assay parameters to adjust the sensitivity window

**Fig. 2:**
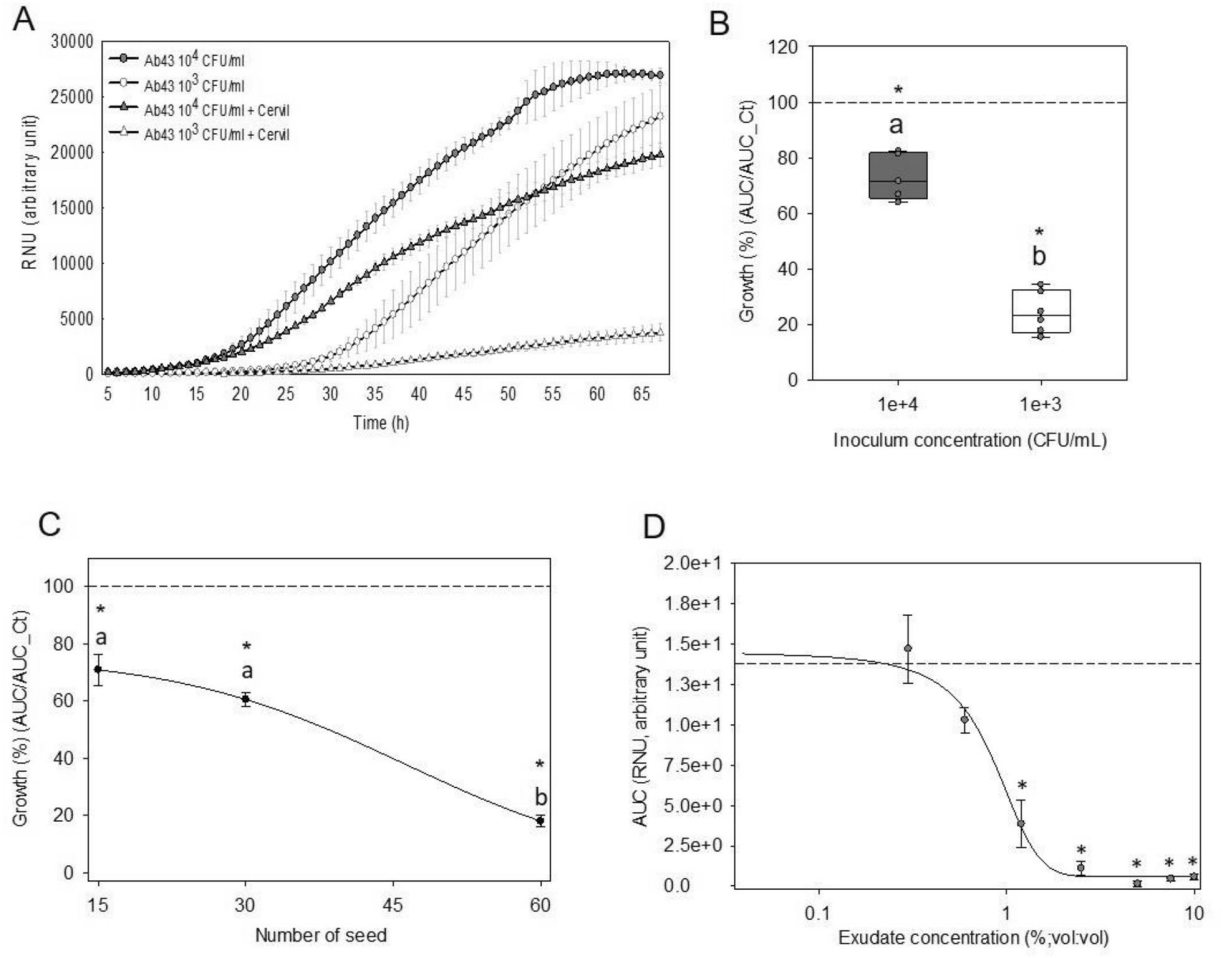
Modulation of assay sensitivity the detection of antimicrobial activity from exudates of primary dormant Cervil seeds. **A** Growth curve of *A. brassicicola* at 10^4^ and 10^3^ CFU/mL with and without (control) exudates. Data represent the average of three technical replicates ± SD. **B** Effect of conidia concentration on antimicrobial activity from exudates. Data are expressed as the normalized growth ratio between the AUC with and without exudate. Box plots represent the median of two replicates. Points in the box plots corresponds of the three technical replicates per biological replicates. **C** The effect of seed number used to produce exudate on the growth of *A. brassicicola* at 10^3^ CFU/mL. Data points are expressed as the normalized growth ratio between the AUC with and without exudate and represent the mean of three replicates ± SD. **D** Dose–response curve of exudate dilution on the fungal growth calibrated at 10^3^ CFU/mL. Data points represent the average of three technical replicates ± SD. Data were fitted with a sigmoidal regression as an aid to the eye. The dashed line corresponds to control growth without exudate. A star indicates a significant difference from control without exudate (Mann–Whitney test, p < 0.05). Different letters indicate a significant difference between conditions (Kruskal–Wallis test, Dunn method, p < 0.05). AUC, area under the curve; RNU, relative nephelometry units

The next goal was to evaluate the sensitivity of the assay. A first parameter that was modulated was the amount of *A. brassicicola* conidia present in the assay. Growth curves without exudate obtained with conidia at concentrations of 10^3^ and 10^4^ CFU/mL (Conidia Forming Unit) showed a delay of the onset of exponential growth phase from 15 to 26 h (Fig. 2A). With the exudate, increasing the conidia concentration from 10^3^ to 10^4^ CFU/mL induced a higher fungal growth (Fig. 2A). When these growth curves are compared with their respective control without exudate, the normalized growth was 70% with a conidia concentration of 10^4^ CFU/mL and 20% with 10^3^ CFU/mL (Fig. 2B). Dilution of the inoculum to 10^2^ CFU/mL resulted in a large variation in fungal growth data between repetitions due to the too small amount of conidia, therefore representing the lower sensitivity limit of the assay.

A second parameter that was modulated was the concentration of antimicrobial molecules in the exudate, either by adjusting the number of seeds or by diluting the exudate. Increasing the number of seeds that produced exudate from 15 to 60 in the same volume led to a decrease of the normalized growth from 70 to 20% (Fig. 2C). This decrease was smaller when the seed number was increased from 15 to 30 seeds compared 30 to 60 seeds, suggesting a threshold effect of the AA (Fig. 2C). Dilution of the exudate resulted in a dose-dependent growth reduction of *A. brassicicola*, with a concentration of 1.2% of exudate that was needed to significantly reduce the growth of *A. brassicicola* with a conidia concentration of 10^3^ CFU/mL (Fig. 2D). From the dose–response curve, the growth inhibition concentration of 50% (IC50) was determined at 1% dilution of the exudate solution. The minimum inhibitory concentration was determined at 0.25% of exudate concentration. Altogether, these results show that both fungal and exudate concentration can be adjusted to optimize the detection threshold, so that a wide range of AA can be assessed depending on the fungal pathogen or seed material. In the following experiments, it was decided to calibrate the standard inoculum concentration at 10^3^ CFU/mL, and to use a tenfold diluted exudate solution collected from 60 seeds.

### Genetic diversity in antimicrobial defense of seed exudates

**Fig. 3:**
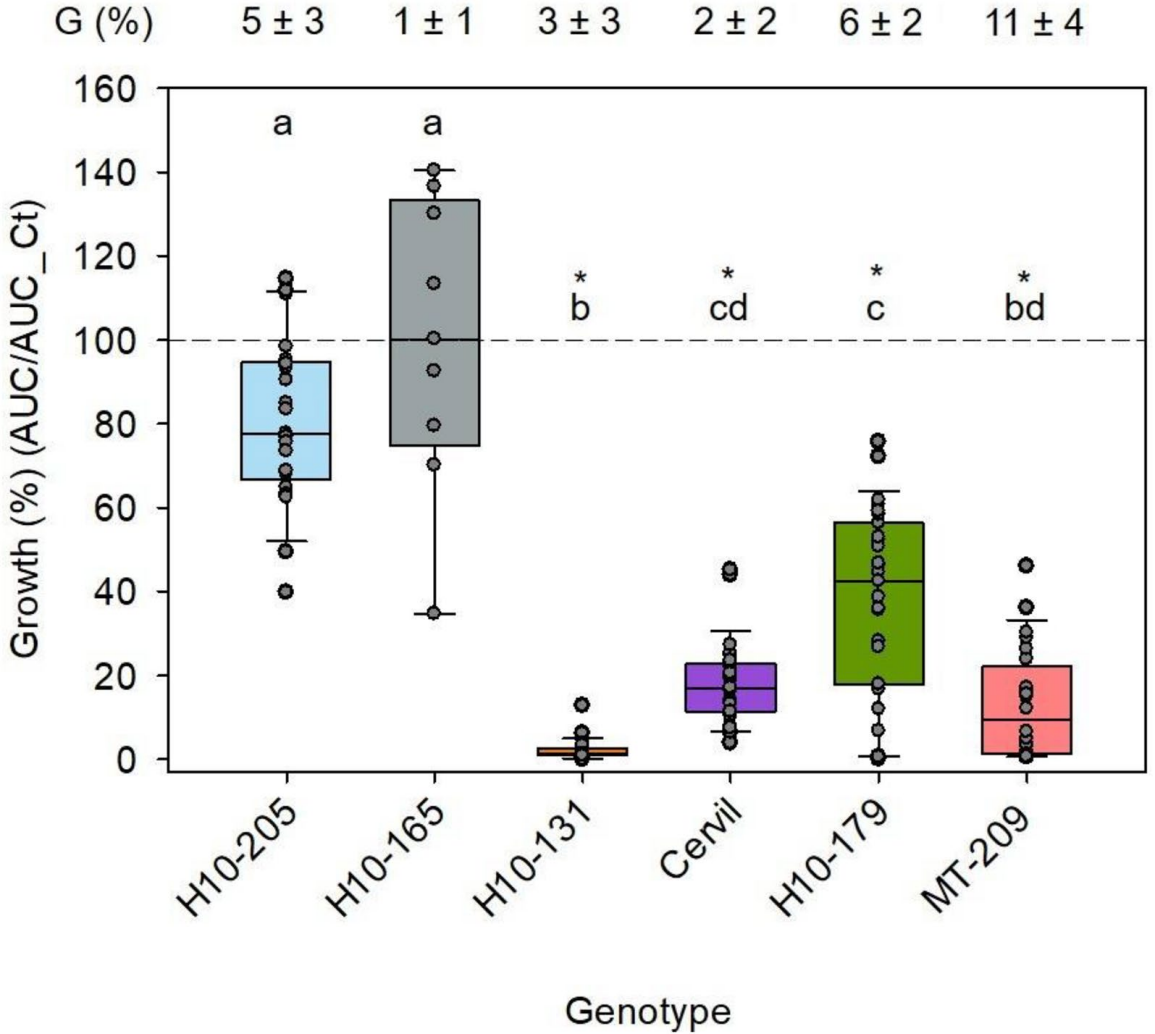
Genetic diversity of antimicrobial activity in exudates of primary dormancy seeds against *A. brassicicola* at 10^3^ CFU/mL. Data are expressed as the normalized growth ratio between the AUC with and without exudate. Points in the box plots corresponds of the three technical replicates per biological replicate (n). n = 9 for H10-131, Cervil and H10-179; n = 8 for MT-209; n = 7 for H10-205 and n = 3 for H10-165.The dashed line corresponds to control growth without exudate. The star indicates a significant difference from control (t-test or Mann–Whitney test, p < 0.05). Different letters indicate a significant difference between genotypes (Kruskal–Wallis test, Dunn method, p < 0.05). G (%), germination percentage determined on three replicates of 60 seeds (± se)

To investigate if there is a genetic variability in AA in the seed exudate, we selected five additional accessions of the MAGIC population with seeds that exhibited primary dormancy. Exudates were produced by imbibing seeds for 5 day and removing the germinated seeds daily to avoid possible artifacts. Residual percentages of germination at harvest of the exudate after 5d of imbibition were less than 11%, and no further increase in germination occurred after 5 day (Fig. 2, Additional file 1: Figure S1). For the six genotypes studied, exudates from H10-205 and H10-165 did not significantly reduce fungal growth, with a normalized growth of 78 and 100% respectively (Fig. 3). Exudates from the other four genotypes significantly decreased fungal growth in a quantitative manner, with normalized growth ranging from 43 to 1% (Fig. 3). A detailed analysis of the growth parameters revealed that the lag phase exhibited significant differences for two genotypes, Cervil and H10-205, compared to control without exudate (Additional file 2: Figure S2A). Values of slope, maximum slope and AUC were significantly lower for exudates from genotypes H10-131, Cervil, H10-179 and MT-209 (Additional file 2: Figure S2B, C). The time to reach the maximum slope was significantly different for H10-205 and H10-179 (Additional file 2: Figure S2D). Overall, these data reveal a genotype-dependent graduation in the AA levels from primary dormant seeds during imbibition.

### Imbibition kinetics of appearance of AA in exudates

**Fig. 4:**
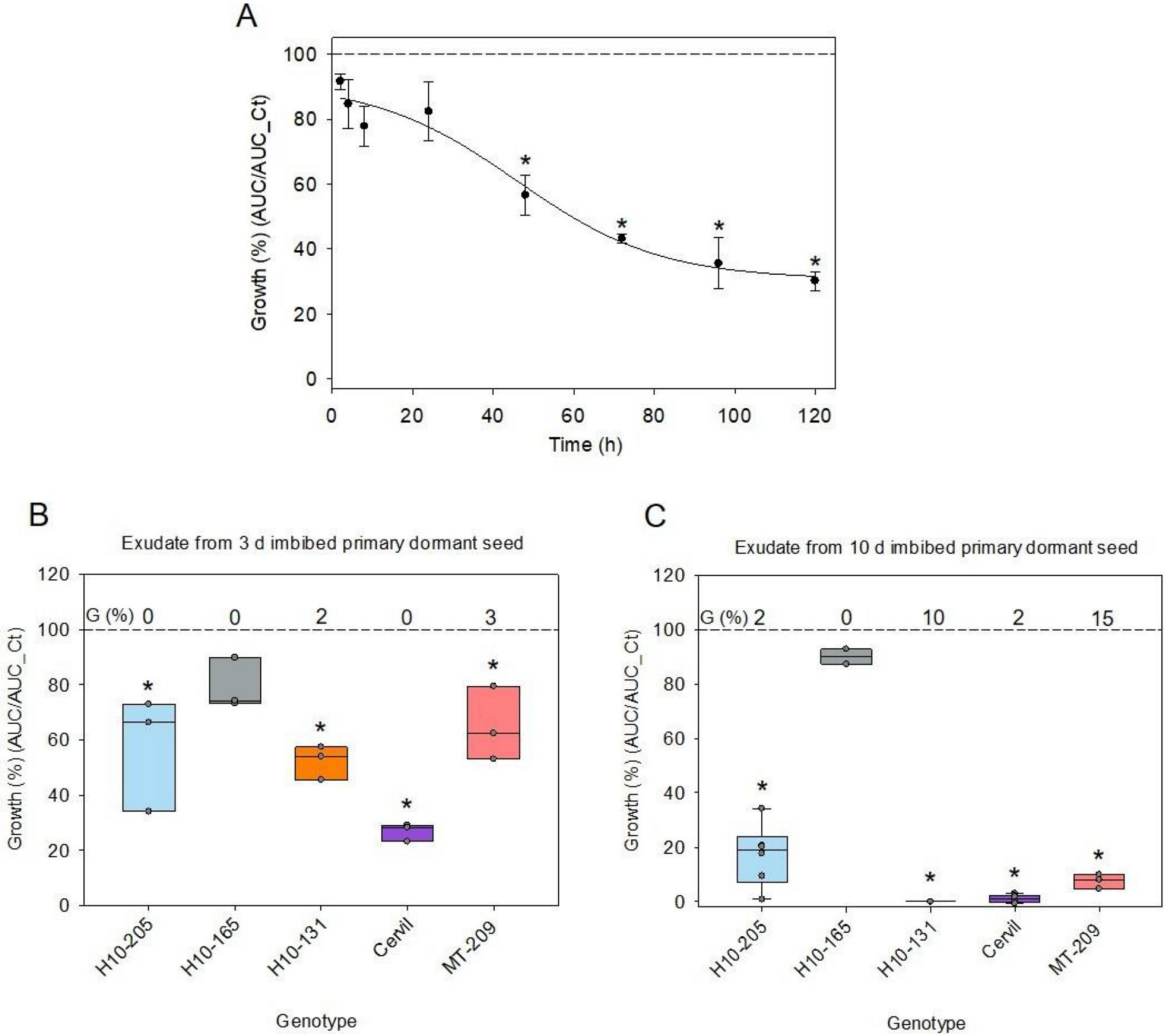
Dynamics of antimicrobial activity during imbibition of primary dormant seeds. **A** The effect of imbibition time when Cervil seed exudate is collected on the growth of *A. brassicicola* at 10^3^ CFU/mL. Data represent the average of three technical replicates ± SD and are fitted with a sigmoidal regression as an aid to the eye. **B**, **C**. The impact of the genotypes on growth of *A. brassicicola* at 10^3^ CFU assessed after 3 (**B**) and 10 day (**C**) of imbibition before collecting the exudate. Data are expressed as the normalized growth ratio between the AUC with and without exudate. Points in the box plots corresponds to three technical replicates per biological replicate (n). n = 1 for data in the panel B. In the panel C, n = 1 for H10-165, H10-131 and MT-209; n = 2 for H10-205 and Cervil. The dashed line corresponds to control growth without exudate. A star indicates a significant difference from control (t-test or Mann–Whitney test, p < 0.05). G (%), germination percentage was determined on 60 seeds

To obtain the kinetics of release of molecules responsible for AA, exudates were collected at different time points during imbibition of Cervil seeds. The AA in the exudate resulting in decreased fungal growth followed a sigmoidal pattern during seed imbibition (Fig. 4A). Exudates harvested within 24 h of imbibition did not significantly decrease the growth of Ab43. Between 24 and 48 h, fungal growth was gradually inhibited, with a significant effect at 48 h equivalent to 57% of the AUC of the control without exudate (Fig. 4A). Further imbibition for 96 and 120 h reduced the normalized growth to 35 and 30%, respectively.

Considering the differences in AA of exudates between genotypes (Fig. 3), we next investigated if these differences could be explained by the kinetics of exudation during imbibition. The corresponding germination curves are shown in Additional file 3: Figure S3. When imbibition was reduced to 3 day of imbibition, significant differences compared to the control without exudate were evident for all genotypes (Fig. 4B). Comparison of AA between exudate recovered after 3 and 5 day of imbibition shows that the AA increased for all genotypes except H10-205 and H10-165 (compare Fig. 4B with Fig. 3). Further increasing the imbibition time to 10 day before collecting the exudate showed almost total growth reduction for the exudates of all genotypes except for H10-165 (Fig. 4C). This reduction was the most important for H10-205. Nonetheless, higher inoculum at 10^4^ CFU/mL using 10 day exudates confirmed the quantitative differences in AA among the different genotypes in a comparable order to that found with 10^3^ CFU/mL at 5 day (Additional file 4: Fig. S4). Thus, both the kinetics of release and amount of molecules leading to AA is strongly genotype-dependent, with one genotype (H10-165) showing no detectable AA in the seed exudate, even after prolonged incubation.

### Evolution of antimicrobial activity during thermodormancy

**Fig. 5:**
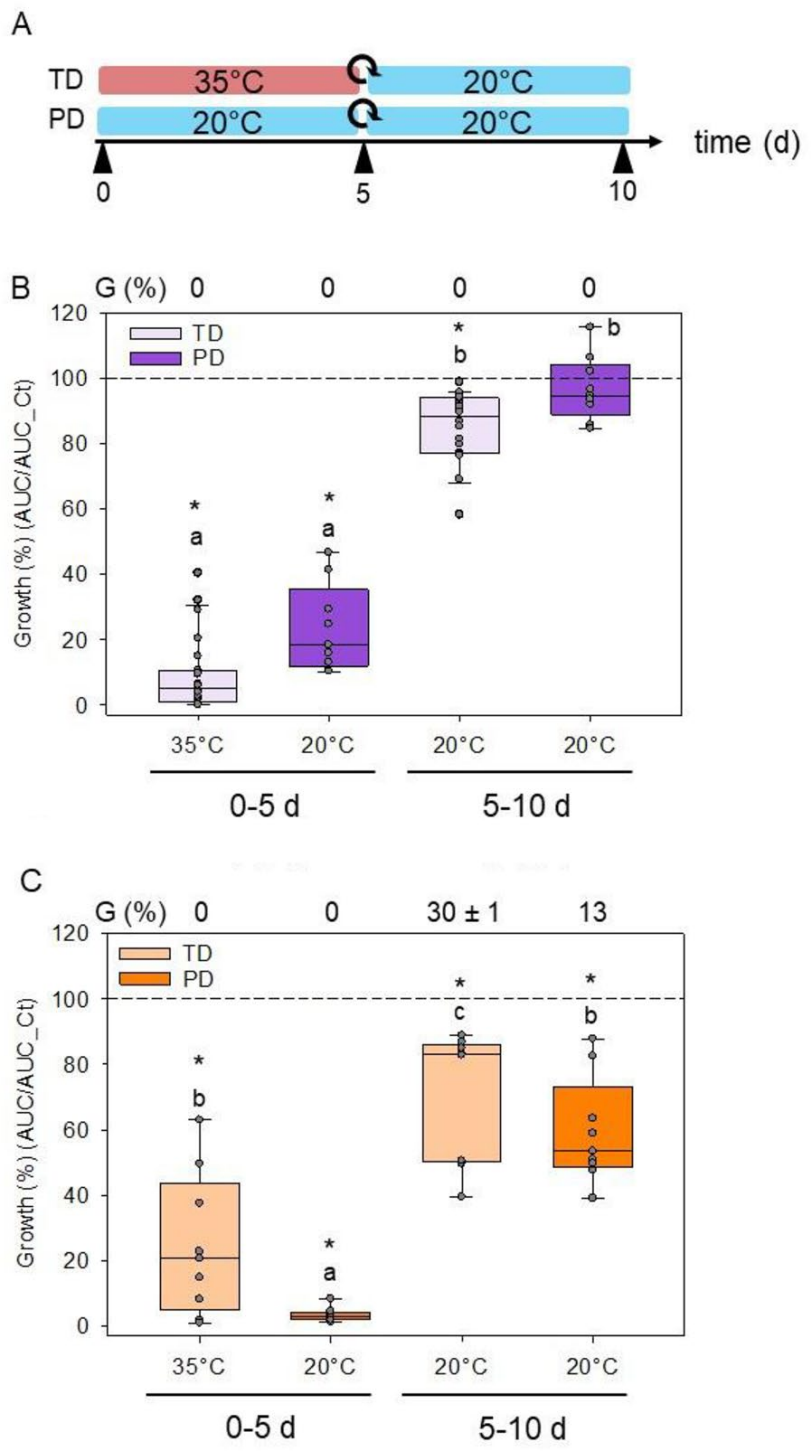
Influence of primary and secondary dormancy on antimicrobial activity. **A** Experimental design used to produce the exudate. Thermodormancy (TD) was induced by a 5 day imbibition at 35 ℃ in the dark using non dormant seeds. Exudates were collected after 5 d of imbibition, seeds were transferred into a fresh water solution and exudate was collected after another 5 d of incubation at 20 ℃. The rolled-up arrow corresponds to the replacement of the exudate by fresh water. B-C. Normalized growth of *A. brassicicola* calibrated at 10^3^ CFU/mL of primary dormant (PD) and thermodormant seeds of the Cervil (**B**) and H10-131 (**C**) genotype after 0–5 and 5–10 d of incubation. Data are expressed as the normalized growth ratio between the AUC with and without exudate. Points in the box plots corresponds of the three technical replicates per biological replicate (n). n = 8 for TD Cervil at 35 ℃; n = 6 for TD Cervil at 20 ℃ and n = 3 for the others conditions. The dashed line corresponds to control growth without exudate. A star indicates a significant difference from control (t-test or Mann–Whitney test, α = 0.05). Different letters indicate a significant difference between conditions from Cervil genotype (Kruskal–Wallis test, Dunn method, p < 0.05) and H10-131 genotype (ANOVA, Holm-Sidak method, p < 0.05). G (%), germination percentage was determined on three replicates of 60 seeds (± se)

In tomato, secondary dormancy can be induced in non-dormant seeds by a 5 day imbibition at 37 ℃ [8]. This protocol was used to induce thermodormancy in seeds of Cervil and H10-131, except that seeds were incubated at 35 ℃. No germination was observed during the heat imbibition treatment, and upon return at 20 ℃, Cervil and H10-131 exhibited 0% and 30% of germination, respectively (Fig. 5). Next, we investigated whether the exudate produced during induction of thermodormancy exhibited AA. For H10-131, the few germinated seeds (Fig. 5) were removed daily to keep only thermodormant seeds in the exudate. Exudates collected after 5 day of imbibition at 35 ℃ displayed a strong AA, with a normalized growth of 5 and 20% for Cervil and H10-131, respectively (Fig. 5B, C). These values are comparable to exudate obtained for primary dormant seeds imbibed at 20 ℃ (Fig. 5B, C).

Since a longer imbibition of 10 day increased the AA level of the exudate (Fig. 4C), we tested whether thermodormant seeds continued to produce an exudate with AA upon return to 20 ℃. For this purpose, primary and thermodormant seeds were transferred to a new solution at 20 ℃ and incubated for another 5 day, after which exudate was harvested (Fig. 5A). In Cervil seeds, no AA was observed after this second incubation at 20 ℃ in the exudate of primary dormant seeds and was strongly reduced for thermodormant seeds (Fig. 5B). No significant difference was observed when the type of dormancy was compared (Fig. 5B). In H10-131, AA was still present in the exudate of both primary and thermodormant seeds after the second incubation, although significantly less than that of the first imbibition (Fig. 5C). This indicates that most of the compounds exhibiting AA are extruded during the first 5 day of seed imbibition but the kinetics depends on the genotype. Primary dormant seeds that were imbibed for 5 day, dried to their original water content, and subsequently being re-imbibed for 5 day produced exudates with no AA (Additional file 5: Figure S5), suggesting that the appearance of AA is not linked to temporal solute leakage that is related to the imbibitional damage [40].

### Antimicrobial activity in germinated seeds and seedlings

**Fig. 6:**
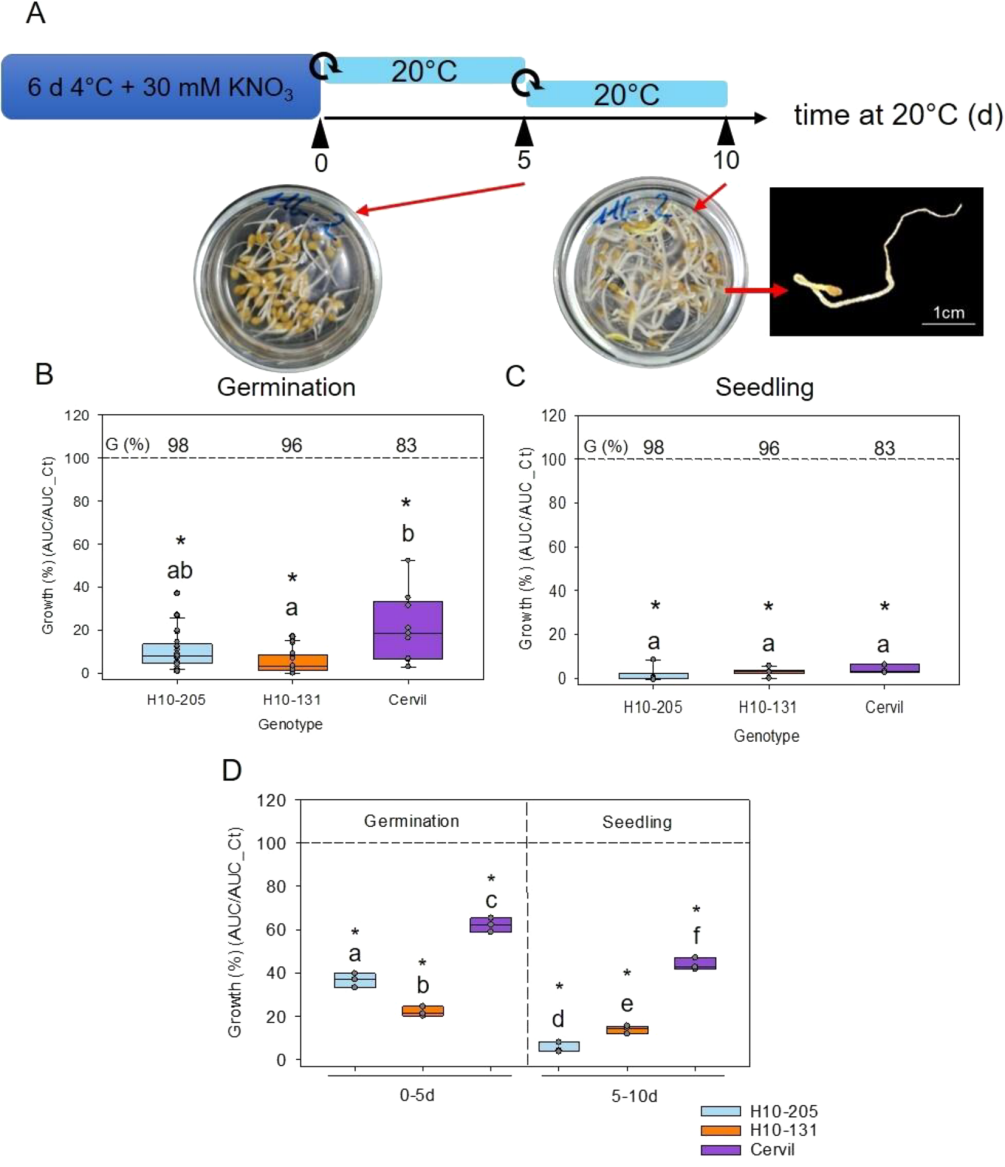
Evolution of antimicrobial activity during germination and seedling growth. **A** Experimental design used to release dormancy and produce exudates after germination (5 d) and seedling growth (10 d). The rolled-up arrow corresponds to the replacement of the exudate by fresh water. **B**, **C**. Antimicrobial activity of exudates during germination seeds (**B**) and during seedling growth (**C**) of H10-205, H10-131 and Cervil genotypes against *A. brassicicola* growth calibrated at 10^3^ CFU/mL. D. Antimicrobial activity from seed exudate during germination and seedling growth against *A. brassicicola* calibrated at 10^4^ CFU/mL. Data are expressed as the normalized growth ratio between the AUC with and without exudate. Points in the box plots corresponds of the three technical replicates per measurement. Measurements of antimicrobial activity of the exudate from H10-205, H10-131 and Cervil genotype were repeated respectively seven, three and five times in the panel **B**; respectively four, one and two times in the panel **C**; and one time for all genotype in the panel **D**. The dashed line corresponds to control growth without exudate. The star indicates a significant difference from control (t-test or Mann–Whitney test, p < 0.05). Different letters indicate a significant difference between genotypes (Kruskal–Wallis test, Dunn method, p < 0.05). G (%), germination percentage was determined on 60 seeds

Next, we investigated whether the exudates leaking from non-dormant seeds during imbibition also exhibits AA. Primary dormancy was released by incubating dormant seeds in 30 mM KNO_3_ at 4 ℃ for 6 day [37]. Thereafter, seeds were transferred to sterile water at 20 ℃ for 5 d and exudate was harvested. At this time point, seeds of all genotypes had germinated between 83 and 95% (Fig. 6A). Exudates from the germinated seeds led to a strong reduction in fungal growth with normalized growth ranging from 3 to 15% according to the genotype (Fig. 6B). A KNO_3_ solution at a concentration similar that of the exudate had no effect on the growth of *A. brassicicola* without exudate (Additional file 6: Figure S6). Likewise, imbibition of seeds that were naturally afterripened by 8 months at 20 ℃ in the dark also resulted in strong AA in the exudates of the germinated seeds (Additional file 7: Figure S7).

To determine the AA at a later stage of seedling development, germinated seeds were transferred to a new solution and incubated for another 5 day at 20 ℃ before collecting the exudate. During this period, germinated seeds had developed into seedlings with a longer radicle and hypocotyl and cotyledons that had emerged from the seed coat (Fig. 6A). At this stage, there was no fungal growth with the exudates from all genotypes (Fig. 6C), indicating a very strong AA. This was even true for the exudate from genotype H10-205 which showed a weak AA in the exudate of primary dormant seeds (Fig. 3). An inoculum concentration of 10^4^ CFU/mL also led to a significant growth inhibition (Fig. 6D). This higher concentration allowed for a better discrimination in the AA between the different genotypes, and showed that exudate from seedlings of H10-205 showed the strongest AA of all three genotypes tested (Fig. 6D). These results demonstrate that different developmental stages from dormant seed to seedlings show different levels of AA in the exudate that vary between genotypes, highlighting a genetic diversity x developmental stage interaction in defense.

### Antimicrobial activity against a host pathogen, *Alternaria alternata*

**Fig. 7:**
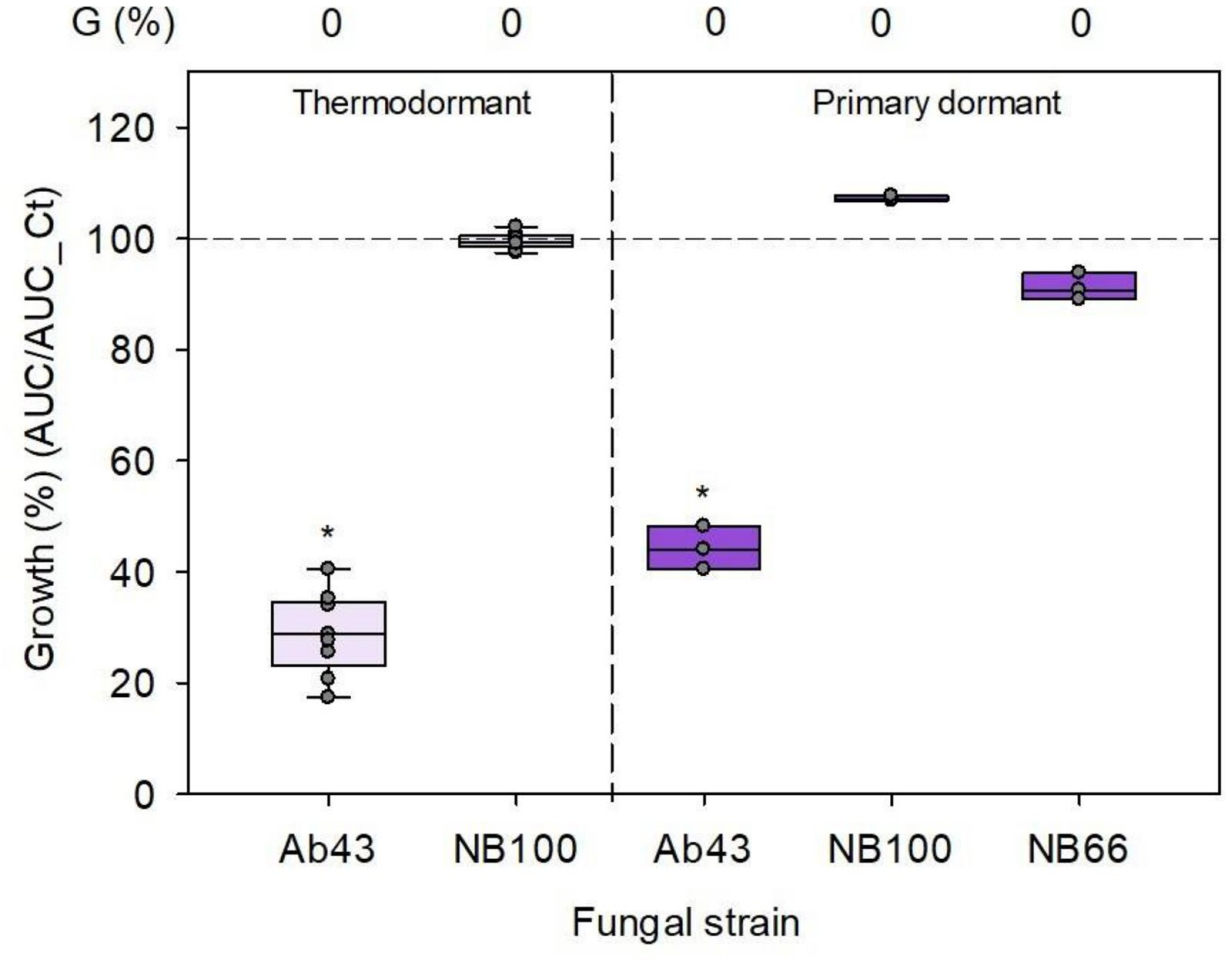
Influence of exudate from dormant Cervil seeds on growth of *A. brassicicola* and *A. alternata*. The antimicrobial activity of exudates was measured by nephelometry on *A. brassicicola* and *A. alternata* growth calibrated at 10^3^ CFU/mL. Data are expressed as the normalized growth ratio between the AUC with and without exudate. Points in the box plots correspond to three technical replicates per biological replicate (n). n = 3 for thermodormant Cervil and n = 1 for primary dormant Cervil. The dashed line corresponds to control growth without exudate. A star indicates a significant difference from control (t-test or Mann–Whitney test, α = 0.05). G (%), germination percentage was determined on 60 seeds

The strain of *A. brassicicola* used in this study is a pathogen with a wide host range, mostly in the Brassica genus [41]. A pathogenicity test of Ab43 on tomato seedlings was performed and confirmed the weak pathogenicity of this strain as 97% of the population showed little or no symptoms (Additional file 8: Figure S8). In contrast, inoculation with known tomato pathogens *A. alternata* strains NB100 and NB66 led to 68 and 56% of seedlings exhibiting severe symptoms (Additional file 8: Figure S8). Exudates of primary and thermodormant seeds from Cervil did not impact the growth of NB100 and NB66 strains, while they repressed growth of Ab43 (Fig. 7). This confirms that tomato seed exudates exhibit a nonhost resistance against filamentous fungi.

## Discussion

Nephelometry allowed us to quantify the Ab43 growth reduction by seed exudates, and provided several growth parameters that are difficult to obtain using conventional bioassays. These parameters are powerful indicators to understand how a compound affects fungal behavior. Indeed, the growth of a fungus can be inhibited either by the inhibition of conidia germination or by a reduction of mycelial hyphae growth. Such information is therefore useful, providing important knowledge to understand the pathogen's life-history traits. Our data show that exudates from primary dormant tomato seeds mainly reduce both the growth rate (i.e., slope, (Additional file 2: Figure S2B) and fungal biomass (i.e., AUC, (Fig. 3), whereas the lag phase does not seem to be strongly impacted (Additional file 2: Figure S2A). For Ab43, it has been experimentally demonstrated that in nephelometry a reduction of fungal biomass reflects a biocidal effect, whereas an increase in lag phase is due to a biostatic effect of the compounds [30]. Thus, in line with these observations, our results warrant further studies to examine whether seed exudates lead to biocidal effects rather than a biostatic effect. Our data show the level AA in the seed exudates decreased with a more concentrated Ab43 inoculum (i.e. 10^4^ CFU/mL) (Fig. 2B). Likewise the minimal inhibitory concentration of amphotericin B increased with increasing fungal inoculum size [42]. In our study, exudates from primary seeds imbibed for 10 d (Fig. 4C) and seedlings (Fig. 6C) exhibited a very strong AA against Ab43 calibrated at 10^3^ CFU/mL. Such concentration did not allow to reveal genotyping differences in contrast to a tenfold more concentrated inoculum (Additional file 4: Figure S4; Fig. 6D). Thus, modulating the inoculum concentration makes it possible to adapt and widen the sensitivity window to better discriminate genotype differences when the AA of exudate is very strong.

Our findings extend previous works showing that seeds leak substances with antimicrobial properties [21, 24, 43, 44]. The AA against Ab43 depends on both the genotype (Fig. 3) and the physiological stage of the seed (Fig. 6). These differences can not be explained by differences in the seed weight. Seeds of the Cervil genotype produced a high AA and yet were the smallest among the genotypes tested, with a 1000-seed weight (TSW) of 1.5 *g*, whereas the TSW ranged from 1.9 to 2.2 *g* for H10-179 and H10-131, respectively.

Several hypotheses could explain the differences in AA among exudates. Possibly, variations in exudate AA could be due to the shape and/or density of trichomes on the seed coat as suggested by Dalling et al. [45]. Indeed, trichomes on the surface of tomato leaves are known to accumulate a large variety of specialized metabolites [46], and their implication against insects [47–49] and fungal pathogens [50] have been demonstrated. Another possibility for the difference in AA could be attributed to differences in the kinetics of release of exudate between seed genotypes. Cervil produced exudate with a high AA already after 3 d of imbibition, with no major increase after a further incubation of 5 d or 10 d. In contrast, H10-205 exhibited a very slow kinetics of extrusion, with a reduction of fungal relative growth to only 58% after 3 d, but decreasing to a further 19% upon further incubation to 10 d (Figs. 3 and 4). The mechanisms underlying this kinetic effect remain to be determined. It could be due to differences in speed of exudate diffusion, either passive due to genotype-dependent seed coat permeability, as previously observed by Mouden et al. [51], or active, via differential activation of the compound transporters. Differences could be due to different levels of synthesis of antimicrobial compounds throughout seed imbibition that accumulate in the exudate. The AA of exudates from Cervil and H10-131 genotypes in both primary and secondary dormant seeds was high during the first 5 d of imbibition, whereas this activity decreased strongly when the 5 day-old imbibition medium was replaced by water and seeds incubated for another 5 d (Fig. 5). Likewise, a drying seeds after 5 day of imbibition lead to a disappearance of AA during the second imbibition. This suggests that the appearance of exudate AA is not a continuous but a transient phenomenon, and suggests that a reservoir of antimicrobial compounds is produced during seed development and/or imbibition. However, the AA of exudates from primary dormant seeds was higher after 10 d of continuous imbibition (Fig. 4) than two consecutive 5 d periods interrupted by replacing water or drying, suggesting, on the contrary, a continuous appearance of AA in the exudate. The mechanism explaining this effect need further study.

Exudates from germinating seeds are known to contain several defense-related proteins and some of them have antifungal properties [20, 52–54]. Here, we show that the exudates produced after germination and during seedling growth have an AA that is higher than from dormant seeds, especially for the genotype H10-205 (Figs. 3 and 6), suggesting that after germination and during seedling growth, molecules with AA are de novo synthesized. This difference in AA between dormant seeds and seedlings could be due to greater exudation from the seed following protrusion of the radicle or from the growing root during seedling establishment. In radish seed exudates, small cysteine-rich antifungal proteins were found to be preferentially released after rupture of the seed coat [52]. Our data corroborate the observation that the release of compounds by the seeds from imbibition to radicle emergence and seedling growth is not a steady-state process [55]. Indeed, upon the 1st h of imbibition, a massive leakage of compounds takes place, followed by a second leakage after radicle emergence. It should be noted that in our study, the exudate recuperated from the 1st h of imbibition did not contain detectable AA against *A. brassisicola*. In tomato, the amount of sugars in the exudate increases 2.6- and tenfold from seed to seedling to plant root respectively [56]. An intriguing question is whether the type of defense responses or origin of molecules with AA are the same in dormant seeds compared to seedlings. A comparative analysis of the nature of the exudates of dormant seeds and seedlings will be needed to understand the defense mechanisms between dormant seeds and seedlings. In *A. thaliana*, succession of different defenses mechanisms against Ab43 is set in place from imbibition until seedling stage [57].

In this study we demonstrated that seed exudates had an antifungal effect against a nonhost fungus of tomato, *A. brassicicola*, but not against a host fungus, *A. alternata* (Fig. 7). *A. alternata* is able to detoxify α-tomatine, thereby counteracting tomato defenses [58]. In maize, the seed exudate also contributed to the nonhost resistance against *Phytophtora sojae* by inhibiting the chemotaxis signal of the zoospores [26]. Altogether, seed defenses appear to contribute to the nonhost resistance of a plant species through the production of exudate at an early stage of its development. Nonhost resistance is apparently the result of a lack of adaptation of pathogens to plant species. As such, the characterization of AA in seeds exudates and the identification of antimicrobial compounds is a promising lever to understand the nonhost resistance in plants and to seek for novel bioactive molecules with potential applications in biocontrol strategies.

## Conclusions

We developed a nephelometric method to detect and quantify the level of seed defence in exudates during imbibition. The data suggest that the exudate AA is of biocidal nature. Differences in AA were observed among dormant genotypes as well as between different stages of seed germination and seedling development. Thus, according to the physiological status during the early phase of stand establishment from dormancy to seedling emergence, protective barriers are modulated to prevent potential attacks by pathogens. This contributes to the persistence of the seed in the soil and the success of seedling establishment, ensuring the survival of the species.

### Supplementary Information

**Additional file 1:** **Figure S1.** Germination curves of primary dormant tomato seeds during two successive 5 d imbibition periods at 20°C in the dark. Points represent the germination percentage ± standard error of three replicates of 60 seeds (only two for genotype H10-165). The rolled-up arrow corresponds to the replacement of the exudate by fresh water. Imb., imbibition.

**Additional file 2:** **Figure S2.** Genetic diversity in antimicrobial activity of primary dormant seed exudates on the *A. brassicicola* development at 10^3^ CFU/mL. A. Lag phase is calculated as the X-intercept of the regression line obtained during the exponential growth phase. Not determined (NA) for H10-131 seed exudate due to the total inhibition. B. Average slope. C. Maximum slope. D. Time to reach maximum slope. Control corresponds to growth of *A. brassicicola* strain Ab43 without exudate. Points in the box plots correspond to the three technical replicates per biological replicate (n). n=1 for Ab43 (control); n=2 for H10-165 and n=3 for others tested genotypes. A star indicates a significant difference from the control (Mann-Whitney test, p<0.05). RNU, relative nephelometry units.

**Additional file 3:** **Figure S3.** Germination curves of primary dormant tomato seeds from different genotypes during imbibition in water at 20°C in the dark. Points represent the germination percentage and was determined on 60 seeds.

**Additional file 4:** **Figure S4.** The impact of the seed exudate from different genotypes on growth of *A. brassicicola* at 10^4^ CFU/mL, assessed after 10 d of imbibition before collecting the exudate. Data are expressed as the normalized growth ratio between the AUC with and without exudate. Points in the box plots correspond to three replicates. The dashed line corresponds to control growth without exudate. The star indicates a significant difference from control (t-test or Mann-Whitney test, p<0.05). Different letters indicate a significant difference between genotypes (Kruskal-Wallis test, Dunn method, p<0.05). G (%), germination percentage determined on 60 seeds.

**Additional file 5:** **Figure S5.** Effect of exudate from primary dormant seeds of different genotypes on growth of *A. brassicicola* at 10^3^ CFU/mL. Seeds that were first imbibed for 5 d, then rinsed and dried for 2 d at 44% RH, followed by 5d of imbibition in water after which exudate was harvested. A. Experimental design, the exudates were produced during a second 5-day imbibition, corresponding to the blue rectangle after 2 days drying at 43% RH in the dark at 20°C. Data are expressed as the normalized growth ratio between the AUC with and without exudate. Points in the box plots corresponds of the three technical replicates per biological replicates (n). n=2 for H10-165; n=3 for Cervil and n=4 for all others tested genotypes. The dashed line corresponds to control growth without exudate. The star indicates a significant difference from control (t-test or Mann-Whitney test, p<0.05). Different letters indicate a significant difference between genotypes (Kruskal-Wallis test, Dunn method, p<0.05). G (%), germination percentage determined from n replicates of 60 seeds (+/- se).

**Additional file 6:** **Figure S6.** Impact of KNO3 treatment on the growth of *A. brassicicola* at 10^3^ CFU/mL. Area under the curve of *A. brassicicola* (strain Ab43) without (Ct) and with the addition of KNO3 [30mM] diluted to 10%. n=1 and points in the box plots corresponds of the three technical replicates (n). The star indicates a significant difference from control (t-test, p<0.05). No statistical difference was found.

**Additional file 7:** **Figure S7.** Antimicrobial activity of exudate from 8 month after-ripened Cervil seeds during germination and seedling growth against *A. brassicicola* at 10^3^ CFU/mL. Residual dormancy of the seed lots was 32±8%. To produce exudates after germination (0-5 d) and seedling growth (5-10 d) the same experimental design shown in Figure 6 was performed without the 6 d KNO3 treatment. Data are expressed as the normalized growth ratio between the AUC with and without exudate. Points in the box plots corresponds of the three technical replicates per biological replicates (n). n=2 and n=4, respectively for exudates after germination and seedling growth. The dashed line corresponds to control growth without exudate. The star indicates a significant difference from control (t-test or Mann-Whitney test, p<0.05).

**Additional file 8:** **Figure S8.** Disease severity of *A. brassicicola* strain Ab43 and *A. alternata* strains NB100 and NB66 on seedling tomato plant. A. Picture representing the disease severity of tomato seedlings after 10 d of inoculation by either Ab43, NB100, NB66, or water (control). B. Disease severity proportion of tomato seedling against Ab43, NB100 and NB66. Imbibed ungerminated tomato seeds were inoculated by 1µL dropping inoculum of either A. brassicicola strain Ab43 or A. alternata strain NB100 and NB66 calibrated at 10^4^, 10^5^ and 10^6^ CFU/mL. The inoculated seeds were incubated for ten days at 20°C in the light, after which a disease index was assigned to each seedling using a disease scale. 0-1: healthy seedling or very few symptoms; 2-3: small necrosis, partial browning of radicle and/or hypocotyl; 4-5: extensive necrosis, almost or total browning of radicle and/or hypocotyl. The data represent all measurements (n=30) of disease levels without taking into account the concentration factor and are expressed as a proportion (%) of the number of plants assigned to a disease index.

## Data Availability

"The dataset(s) supporting the conclusions of this article is(are) included within the article (and its additional file(s))."
