# Supplementary material for: A method to determine antifungal activity in seed exudates by nephelometry
Source: Plant Methods. 2024 Jan 29;20:16. doi: 10.1186/s13007-024-01144-z (PMC10826049; doi:10.1186/s13007-024-01144-z)
Supplement: Supplementary file 1 — Additional file 1: Figure S1. Germination curves of primary dormant tomato seeds during two successive 5 d imbibition periods at 20°C in the dark. Points represent the germination percentage ± standard error of three replicates of 60 seeds (only two for genotype H10-165). The rolled-up arrow corresponds to the replacement of the exudate by fresh water. Imb., imbibition. [file 13007_2024_1144_MOESM1_ESM.pptx]

## Slide 1
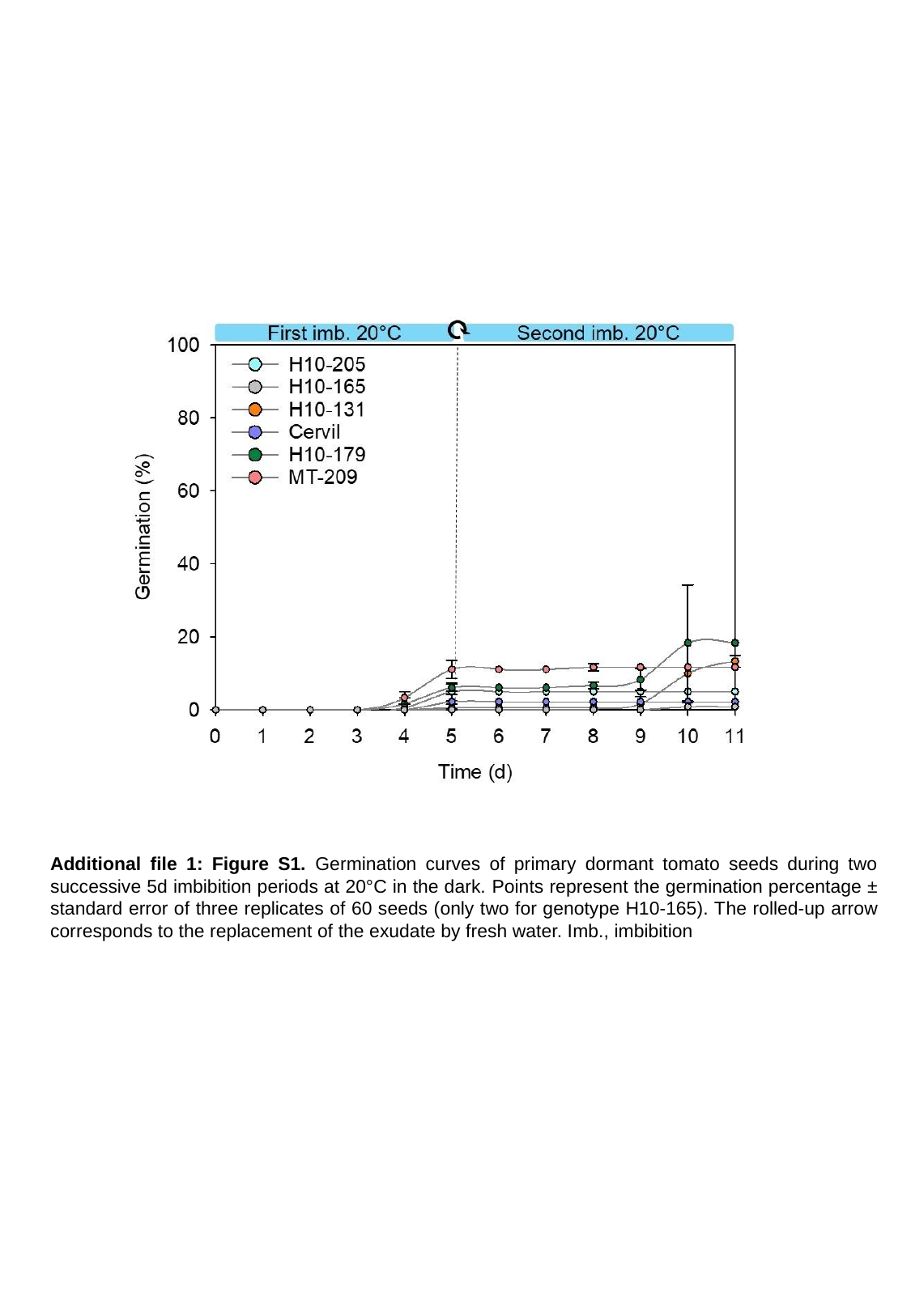

Additional file 1: Figure S1. Germination curves of primary dormant tomato seeds during two successive 5d imbibition periods at 20°C in the dark. Points represent the germination percentage ± standard error of three replicates of 60 seeds (only two for genotype H10-165). The rolled-up arrow corresponds to the replacement of the exudate by fresh water. Imb., imbibition
