# Supplementary material for: A method to determine antifungal activity in seed exudates by nephelometry
Source: Plant Methods. 2024 Jan 29;20:16. doi: 10.1186/s13007-024-01144-z (PMC10826049; doi:10.1186/s13007-024-01144-z)
Supplement: Supplementary file 2 — Additional file 2: Figure S2. Genetic diversity in antimicrobial activity of primary dormant seed exudates on the A. brassicicola development at 103 CFU/mL. A. Lag phase is calculated as the X-intercept of the regression line obtained during the exponential growth phase. Not determined (NA) for H10-131 seed exudate due to the total inhibition. B. Average slope. C. Maximum slope. D. Time to reach maximum slope. Control corresponds to growth of A. brassicicola strain Ab43 without exudate. Points in the box plots correspond to the three technical replicates per biological replicate (n). n=1 for Ab43 (control); n=2 for H10-165 and n=3 for others tested genotypes. A star indicates a significant difference from the control (Mann-Whitney test, p<0.05). RNU, relative nephelometry units. [file 13007_2024_1144_MOESM2_ESM.pptx]

## Slide 1
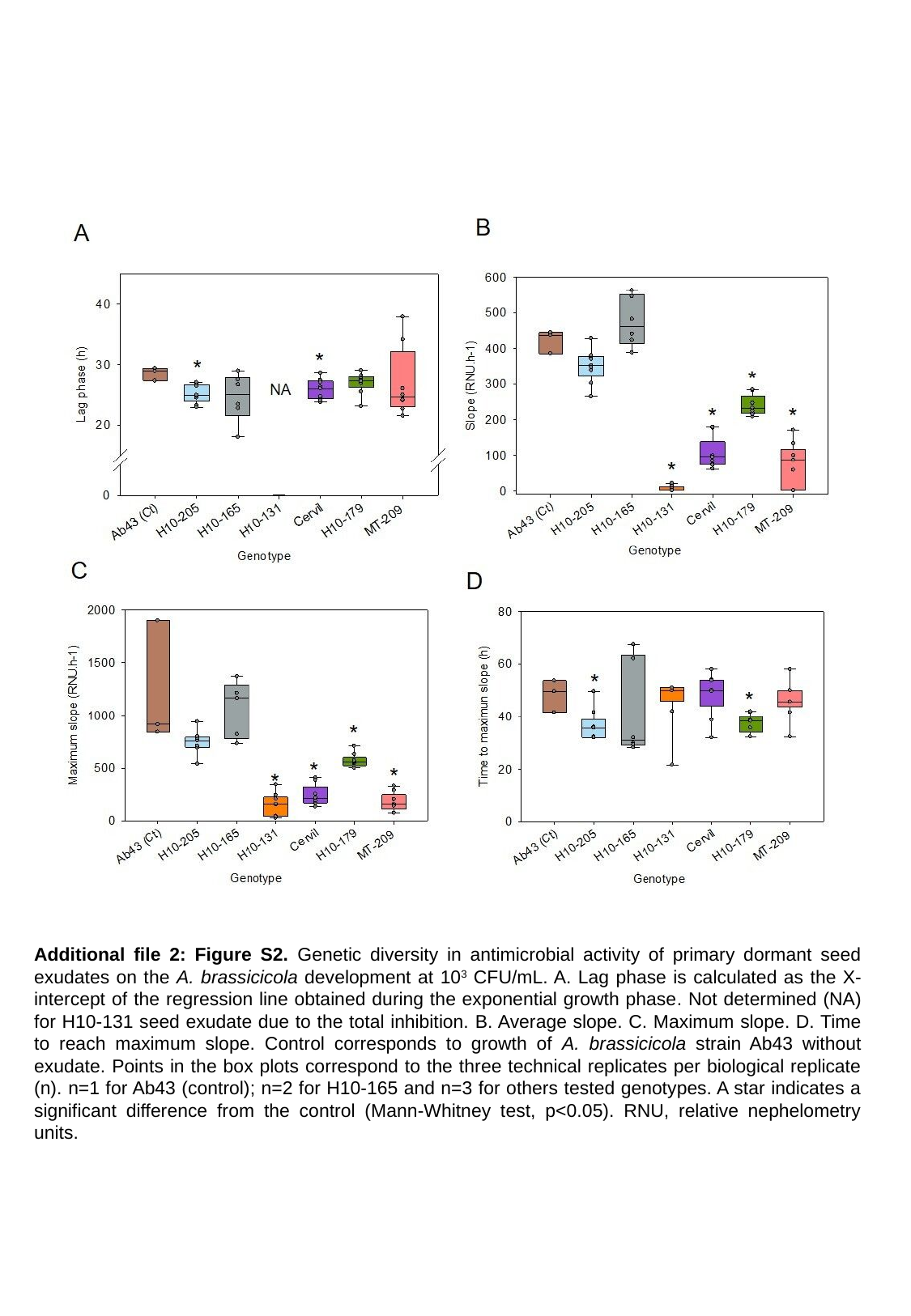

Additional file 2: Figure S2. Genetic diversity in antimicrobial activity of primary dormant seed exudates on the A. brassicicola development at 103 CFU/mL. A. Lag phase is calculated as the X- intercept of the regression line obtained during the exponential growth phase. Not determined (NA) for H10-131 seed exudate due to the total inhibition. B. Average slope. C. Maximum slope. D. Time to reach maximum slope. Control corresponds to growth of A. brassicicola strain Ab43 without exudate. Points in the box plots correspond to the three technical replicates per biological replicate (n). n=1 for Ab43 (control); n=2 for H10-165 and n=3 for others tested genotypes. A star indicates a significant difference from the control (Mann-Whitney test, p<0.05). RNU, relative nephelometry units.
