# Supplementary material for: A method to determine antifungal activity in seed exudates by nephelometry
Source: Plant Methods. 2024 Jan 29;20:16. doi: 10.1186/s13007-024-01144-z (PMC10826049; doi:10.1186/s13007-024-01144-z)
Supplement: Supplementary file 3 — Additional file 3: Figure S3. Germination curves of primary dormant tomato seeds from different genotypes during imbibition in water at 20°C in the dark. Points represent the germination percentage and was determined on 60 seeds. [file 13007_2024_1144_MOESM3_ESM.pptx]

## Slide 1
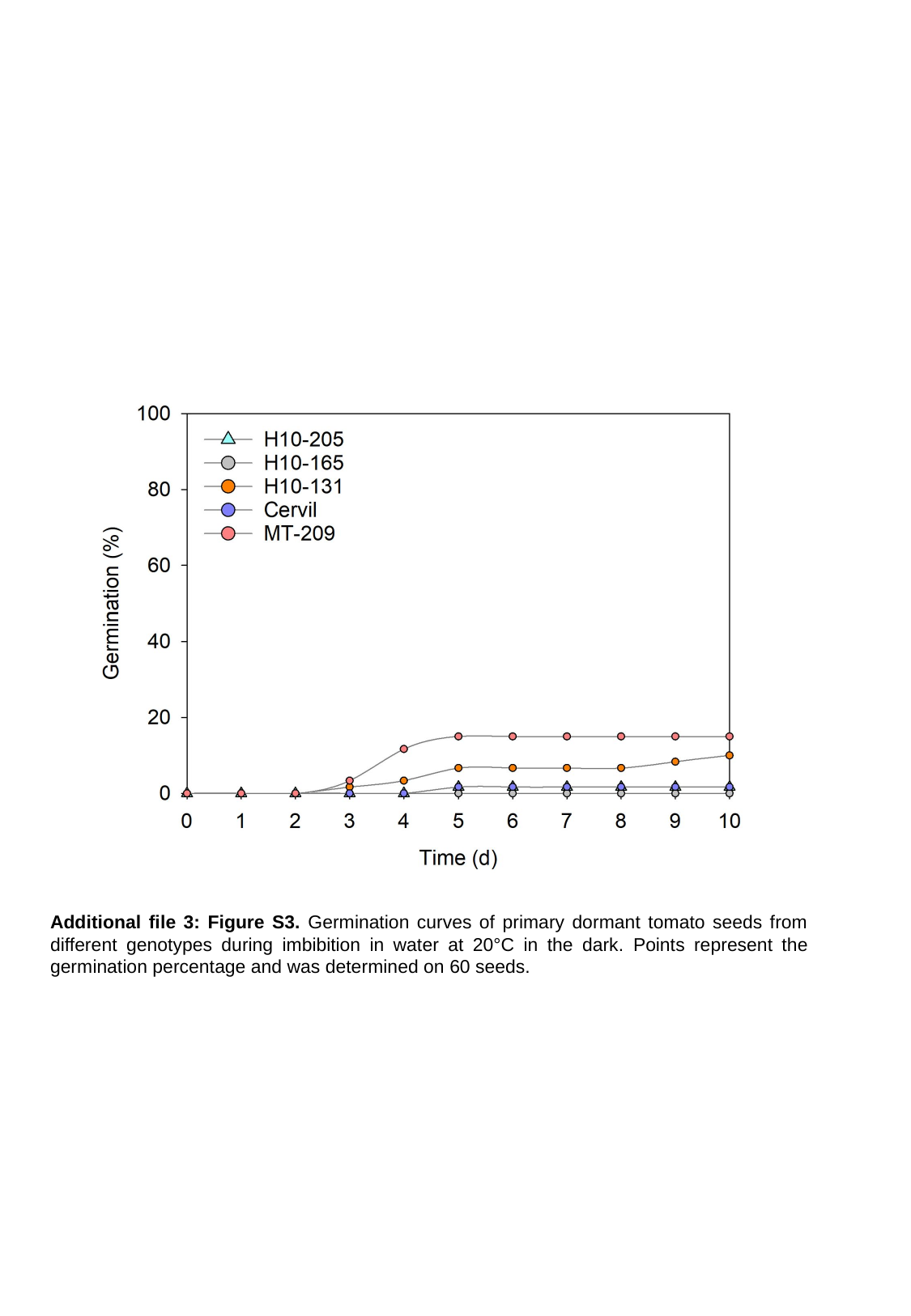

Additional file 3: Figure S3. Germination curves of primary dormant tomato seeds from different genotypes during imbibition in water at 20°C in the dark. Points represent the germination percentage and was determined on 60 seeds.
