# Supplementary material for: A method to determine antifungal activity in seed exudates by nephelometry
Source: Plant Methods. 2024 Jan 29;20:16. doi: 10.1186/s13007-024-01144-z (PMC10826049; doi:10.1186/s13007-024-01144-z)
Supplement: Supplementary file 4 — Additional file 4: Figure S4. The impact of the seed exudate from different genotypes on growth of A. brassicicola at 104 CFU/mL, assessed after 10 d of imbibition before collecting the exudate. Data are expressed as the normalized growth ratio between the AUC with and without exudate. Points in the box plots correspond to three replicates. The dashed line corresponds to control growth without exudate. The star indicates a significant difference from control (t-test or Mann-Whitney test, p<0.05). Different letters indicate a significant difference between genotypes (Kruskal-Wallis test, Dunn method, p<0.05). G (%), germination percentage determined on 60 seeds. [file 13007_2024_1144_MOESM4_ESM.pptx]

## Slide 1
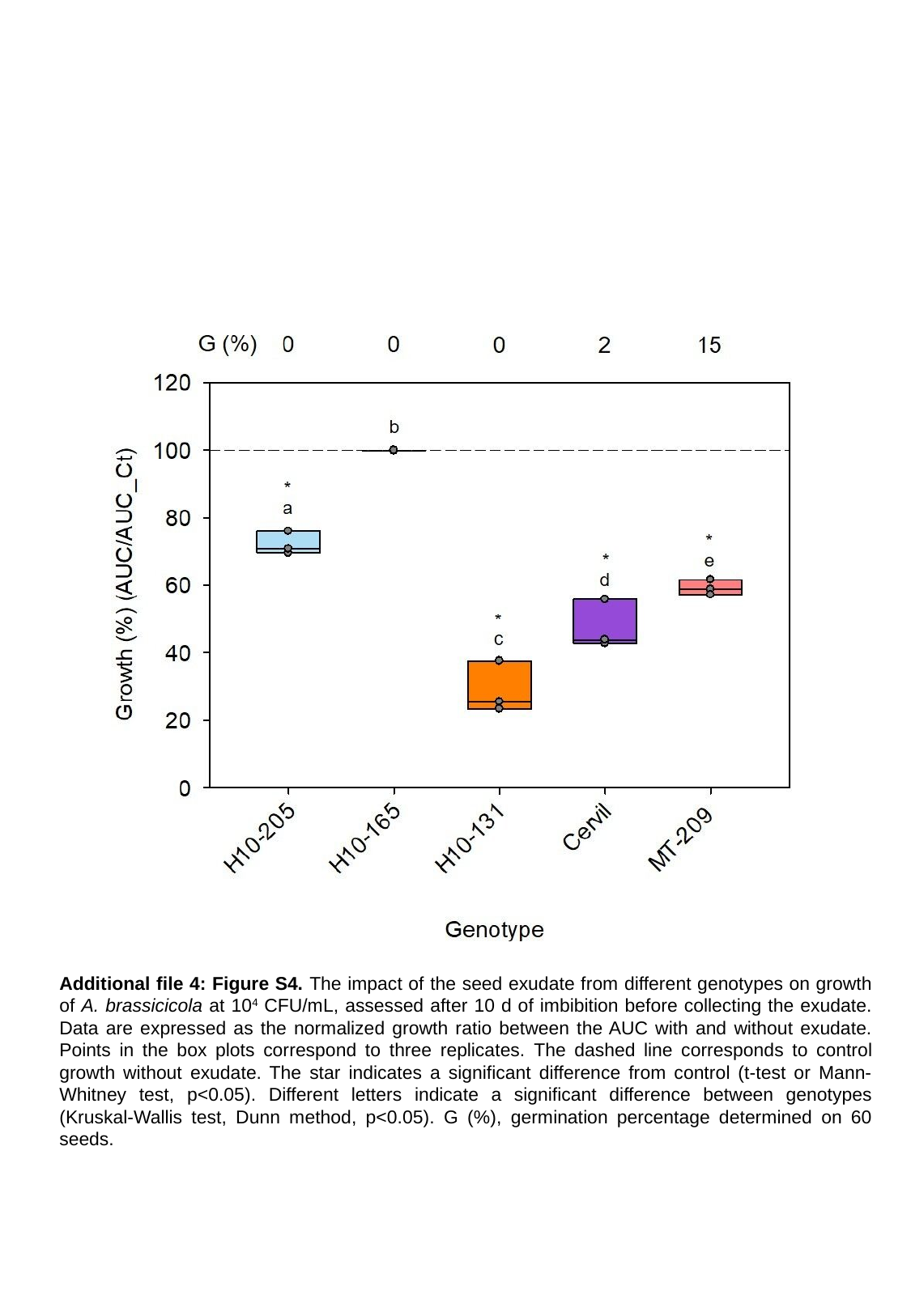

Additional file 4: Figure S4. The impact of the seed exudate from different genotypes on growth of A. brassicicola at 104 CFU/mL, assessed after 10 d of imbibition before collecting the exudate. Data are expressed as the normalized growth ratio between the AUC with and without exudate. Points in the box plots correspond to three replicates. The dashed line corresponds to control growth without exudate. The star indicates a significant difference from control (t-test or Mann-Whitney test, p<0.05). Different letters indicate a significant difference between genotypes (Kruskal-Wallis test, Dunn method, p<0.05). G (%), germination percentage determined on 60 seeds.
