# Supplementary material for: A method to determine antifungal activity in seed exudates by nephelometry
Source: Plant Methods. 2024 Jan 29;20:16. doi: 10.1186/s13007-024-01144-z (PMC10826049; doi:10.1186/s13007-024-01144-z)
Supplement: Supplementary file 5 — Additional file 5: Figure S5. Effect of exudate from primary dormant seeds of different genotypes on growth of A. brassicicola at 103 CFU/mL. Seeds that were first imbibed for 5 d, then rinsed and dried for 2 d at 44% RH, followed by 5d of imbibition in water after which exudate was harvested. A. Experimental design, the exudates were produced during a second 5-day imbibition, corresponding to the blue rectangle after 2 days drying at 43% RH in the dark at 20°C. Data are expressed as the normalized growth ratio between the AUC with and without exudate. Points in the box plots corresponds of the three technical replicates per biological replicates (n). n=2 for H10-165; n=3 for Cervil and n=4 for all others tested genotypes. The dashed line corresponds to control growth without exudate. The star indicates a significant difference from control (t-test or Mann-Whitney test, p<0.05). Different letters indicate a significant difference between genotypes (Kruskal-Wallis test, Dunn method, p<0.05). G (%), germination percentage determined from n replicates of 60 seeds (+/- se). [file 13007_2024_1144_MOESM5_ESM.pptx]

## Slide 1
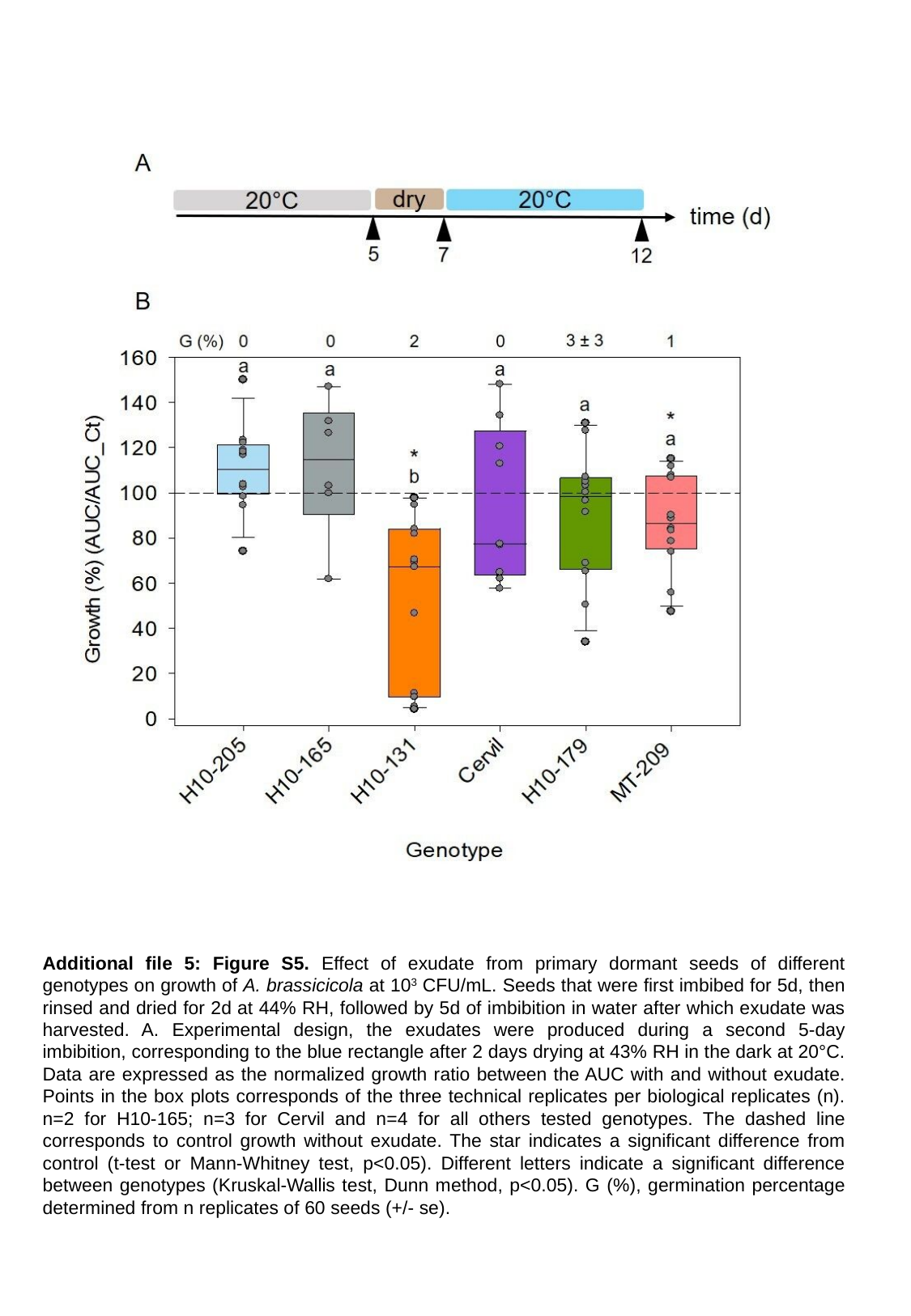

Additional file 5: Figure S5. Effect of exudate from primary dormant seeds of different genotypes on growth of A. brassicicola at 103 CFU/mL. Seeds that were first imbibed for 5d, then rinsed and dried for 2d at 44% RH, followed by 5d of imbibition in water after which exudate was harvested. A. Experimental design, the exudates were produced during a second 5-day imbibition, corresponding to the blue rectangle after 2 days drying at 43% RH in the dark at 20°C. Data are expressed as the normalized growth ratio between the AUC with and without exudate. Points in the box plots corresponds of the three technical replicates per biological replicates (n). n=2 for H10-165; n=3 for Cervil and n=4 for all others tested genotypes. The dashed line corresponds to control growth without exudate. The star indicates a significant difference from control (t-test or Mann-Whitney test, p<0.05). Different letters indicate a significant difference between genotypes (Kruskal-Wallis test, Dunn method, p<0.05). G (%), germination percentage determined from n replicates of 60 seeds (+/- se).
