# Supplementary material for: A method to determine antifungal activity in seed exudates by nephelometry
Source: Plant Methods. 2024 Jan 29;20:16. doi: 10.1186/s13007-024-01144-z (PMC10826049; doi:10.1186/s13007-024-01144-z)
Supplement: Supplementary file 6 — Additional file 6: Figure S6. Impact of KNO3 treatment on the growth of A. brassicicola at 103 CFU/mL. Area under the curve of A. brassicicola (strain Ab43) without (Ct) and with the addition of KNO3 [30mM] diluted to 10%. n=1 and points in the box plots corresponds of the three technical replicates (n). The star indicates a significant difference from control (t-test, p<0.05). No statistical difference was found. [file 13007_2024_1144_MOESM6_ESM.pptx]

## Slide 1
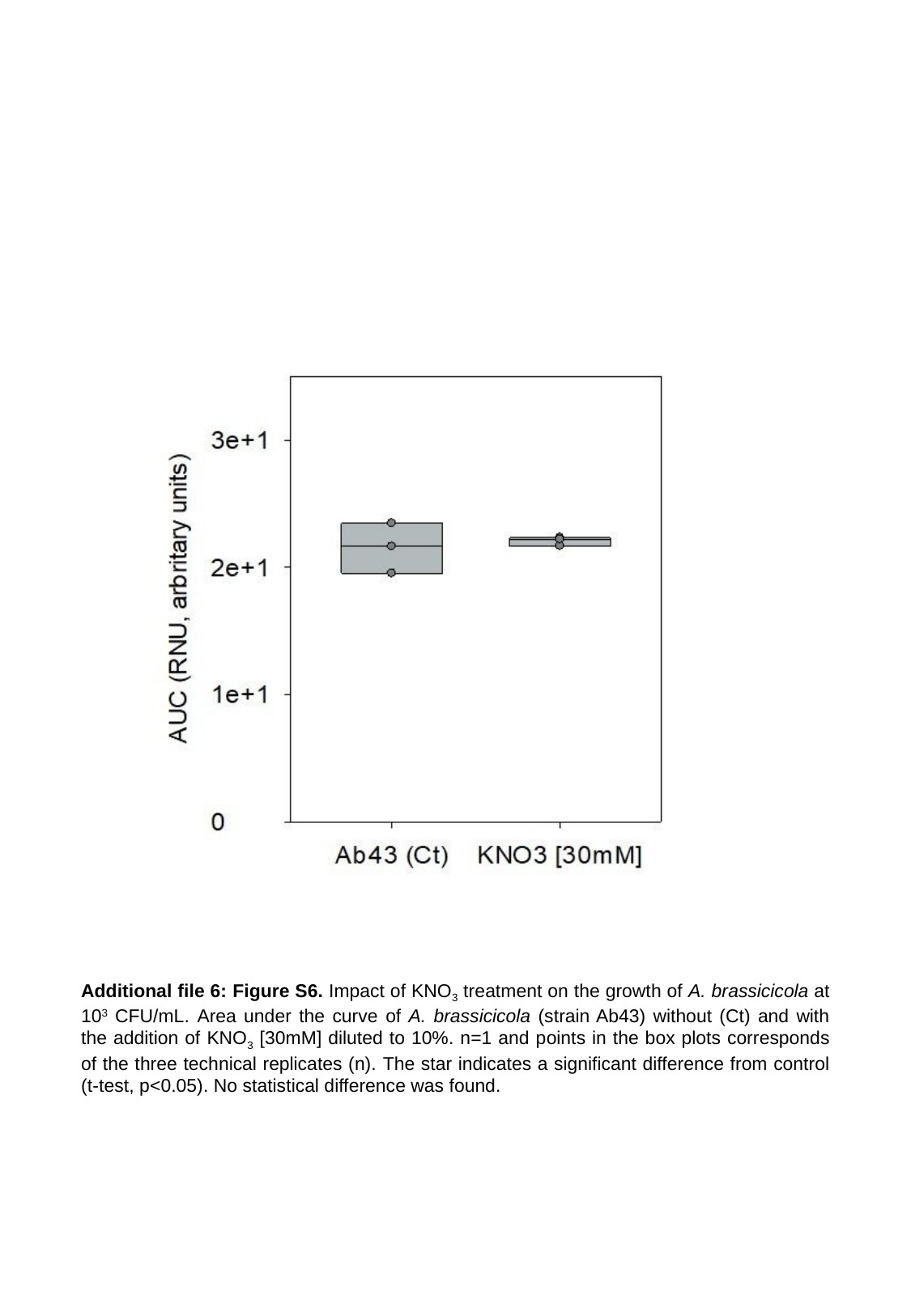

Additional file 6: Figure S6. Impact of KNO3 treatment on the growth of A. brassicicola at 103 CFU/mL. Area under the curve of A. brassicicola (strain Ab43) without (Ct) and with the addition of KNO3 [30mM] diluted to 10%. n=1 and points in the box plots corresponds of the three technical replicates (n). The star indicates a significant difference from control (t-test, p<0.05). No statistical difference was found.
