# Supplementary material for: A method to determine antifungal activity in seed exudates by nephelometry
Source: Plant Methods. 2024 Jan 29;20:16. doi: 10.1186/s13007-024-01144-z (PMC10826049; doi:10.1186/s13007-024-01144-z)
Supplement: Supplementary file 7 — Additional file 7: Figure S7. Antimicrobial activity of exudate from 8 month after-ripened Cervil seeds during germination and seedling growth against A. brassicicola at 103 CFU/mL. Residual dormancy of the seed lots was 32±8%. To produce exudates after germination (0-5 d) and seedling growth (5-10 d) the same experimental design shown in Figure 6 was performed without the 6 d KNO3 treatment. Data are expressed as the normalized growth ratio between the AUC with and without exudate. Points in the box plots corresponds of the three technical replicates per biological replicates (n). n=2 and n=4, respectively for exudates after germination and seedling growth. The dashed line corresponds to control growth without exudate. The star indicates a significant difference from control (t-test or Mann-Whitney test, p<0.05). [file 13007_2024_1144_MOESM7_ESM.pptx]

## Slide 1
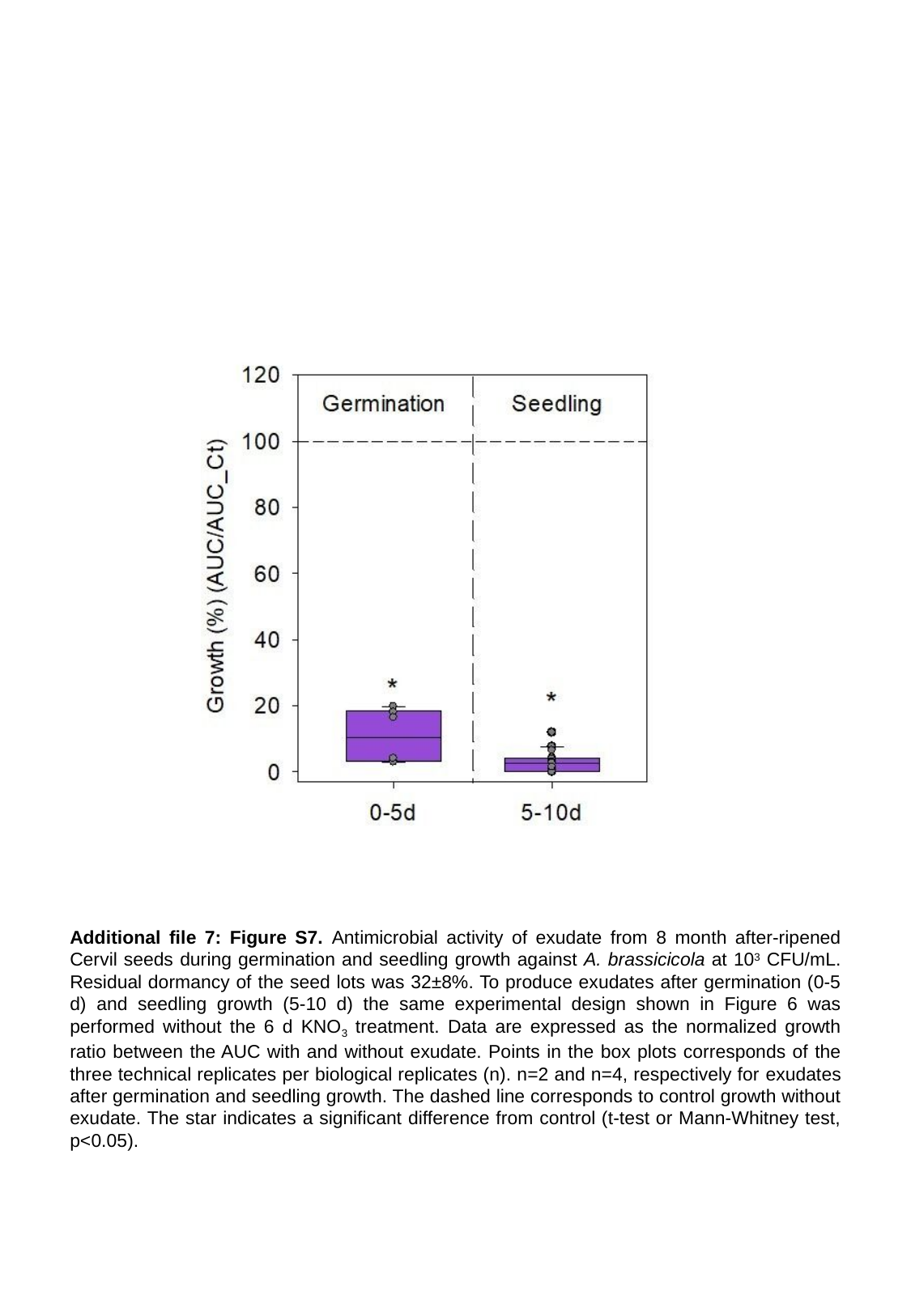

Additional file 7: Figure S7. Antimicrobial activity of exudate from 8 month after-ripened Cervil seeds during germination and seedling growth against A. brassicicola at 103 CFU/mL. Residual dormancy of the seed lots was 32±8%. To produce exudates after germination (0-5 d) and seedling growth (5-10 d) the same experimental design shown in Figure 6 was performed without the 6 d KNO3 treatment. Data are expressed as the normalized growth ratio between the AUC with and without exudate. Points in the box plots corresponds of the three technical replicates per biological replicates (n). n=2 and n=4, respectively for exudates after germination and seedling growth. The dashed line corresponds to control growth without exudate. The star indicates a significant difference from control (t-test or Mann-Whitney test, p<0.05).
