# Supplementary material for: A method to determine antifungal activity in seed exudates by nephelometry
Source: Plant Methods. 2024 Jan 29;20:16. doi: 10.1186/s13007-024-01144-z (PMC10826049; doi:10.1186/s13007-024-01144-z)
Supplement: Supplementary file 8 — Additional file 8: Figure S8. Disease severity of A. brassicicola strain Ab43 and A. alternata strains NB100 and NB66 on seedling tomato plant. A. Picture representing the disease severity of tomato seedlings after 10 d of inoculation by either Ab43, NB100, NB66, or water (control). B. Disease severity proportion of tomato seedling against Ab43, NB100 and NB66. Imbibed ungerminated tomato seeds were inoculated by 1µL dropping inoculum of either A. brassicicola strain Ab43 or A. alternata strain NB100 and NB66 calibrated at 104, 105 and 106 CFU/mL. The inoculated seeds were incubated for ten days at 20°C in the light, after which a disease index was assigned to each seedling using a disease scale. 0-1: healthy seedling or very few symptoms; 2-3: small necrosis, partial browning of radicle and/or hypocotyl; 4-5: extensive necrosis, almost or total browning of radicle and/or hypocotyl. The data represent all measurements (n=30) of disease levels without taking into account the concentration factor and are expressed as a proportion (%) of the number of plants assigned to a disease index. [file 13007_2024_1144_MOESM8_ESM.pptx]

## Slide 1
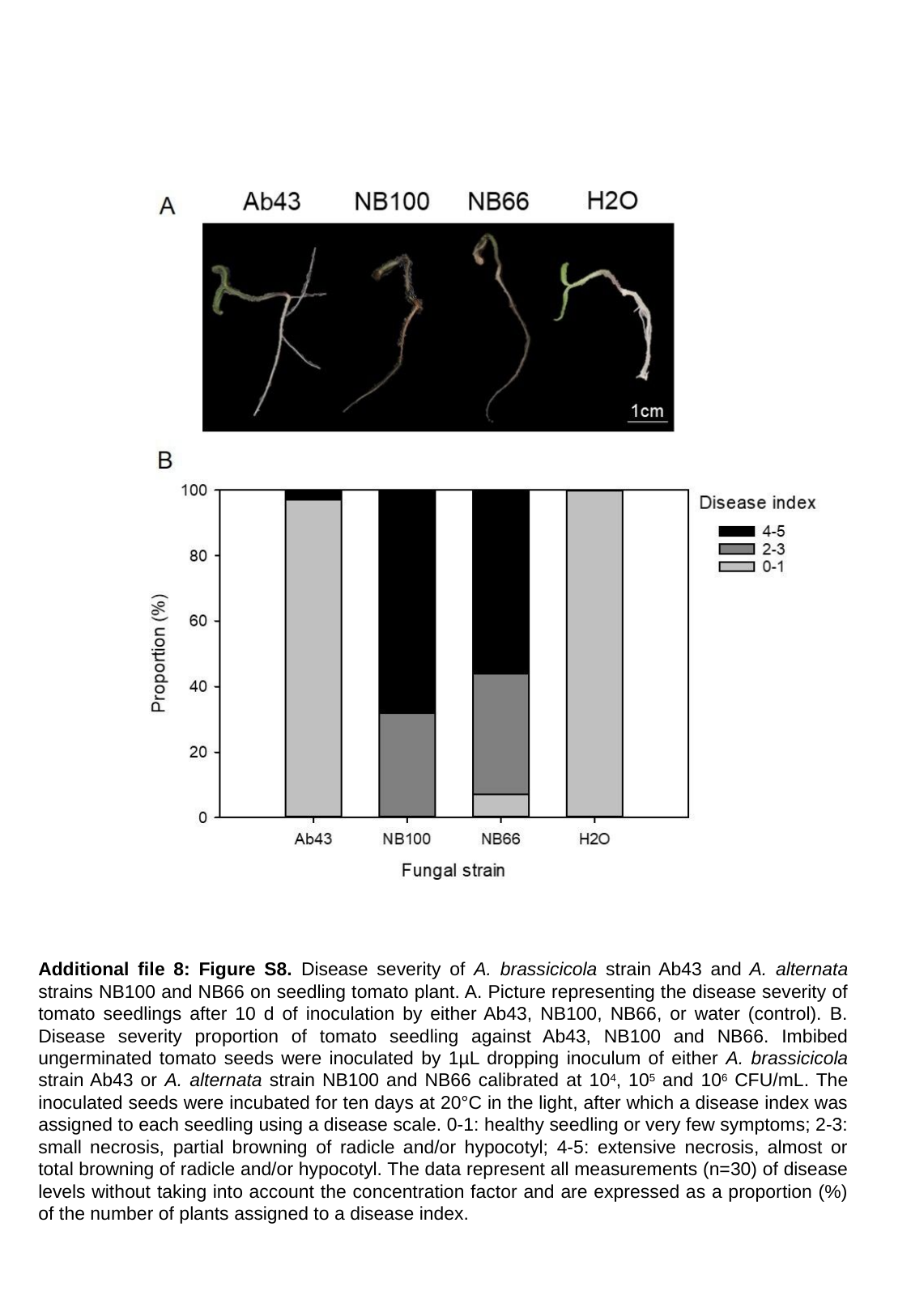

Additional file 8: Figure S8. Disease severity of A. brassicicola strain Ab43 and A. alternata strains NB100 and NB66 on seedling tomato plant. A. Picture representing the disease severity of tomato seedlings after 10 d of inoculation by either Ab43, NB100, NB66, or water (control). B. Disease severity proportion of tomato seedling against Ab43, NB100 and NB66. Imbibed ungerminated tomato seeds were inoculated by 1µL dropping inoculum of either A. brassicicola strain Ab43 or A. alternata strain NB100 and NB66 calibrated at 104, 105 and 106 CFU/mL. The inoculated seeds were incubated for ten days at 20°C in the light, after which a disease index was assigned to each seedling using a disease scale. 0-1: healthy seedling or very few symptoms; 2-3: small necrosis, partial browning of radicle and/or hypocotyl; 4-5: extensive necrosis, almost or total browning of radicle and/or hypocotyl. The data represent all measurements (n=30) of disease levels without taking into account the concentration factor and are expressed as a proportion (%) of the number of plants assigned to a disease index.
